# Fully automated MRI-based convolutional neural network for noninvasive diagnosis of cirrhosis

**DOI:** 10.1186/s13244-024-01872-9

**Published:** 2024-12-12

**Authors:** Tianying Zheng, Yajing Zhu, Yidi Chen, Shengshi Mai, Lixin Xu, Hanyu Jiang, Ting Duan, Yuanan Wu, Yali Qu, Yinan Chen, Bin Song

**Affiliations:** 1 Department of Radiology, West China Hospital, Sichuan University, Chengdu, Sichuan China; 2 SenseTime Research, Shanghai, China; 3 Department of Radiology, Sanya People’s Hospital, Sanya, Hainan China; 4 WCH-SenseTime Joint Lab, SenseTime, Sichuan, China

**Keywords:** Deep learning, Liver cirrhosis, Magnetic resonance imaging, Neural networks, Computer

## Abstract

**Objectives:**

To develop and externally validate a fully automated diagnostic convolutional neural network (CNN) model for cirrhosis based on liver MRI and serum biomarkers.

**Methods:**

This multicenter retrospective study included consecutive patients receiving pathological evaluation of liver fibrosis stage and contrast-enhanced liver MRI between March 2010 and January 2024. On the training dataset, an MRI-based CNN model was constructed for cirrhosis against pathology, and then a combined model was developed integrating the CNN model and serum biomarkers. On the testing datasets, the area under the receiver operating characteristic curve (AUC) was computed to compare the diagnostic performance of the combined model with that of aminotransferase-to-platelet ratio index (APRI), fibrosis-4 index (FIB-4), and radiologists. The influence of potential confounders on the diagnostic performance was evaluated by subgroup analyses.

**Results:**

A total of 1315 patients (median age, 54 years; 1065 men; training, *n* = 840) were included, 855 (65%) with pathological cirrhosis. The CNN model was constructed on pre-contrast T1- and T2-weighted imaging, and the combined model was developed integrating the CNN model, age, and eight serum biomarkers. On the external testing dataset, the combined model achieved an AUC of 0.86, which outperformed FIB-4, APRI and two radiologists (AUC: 0.67 to 0.73, all *p* < 0.05). Subgroup analyses revealed comparable diagnostic performances of the combined model in patients with different sizes of focal liver lesions.

**Conclusion:**

Based on pre-contrast T1- and T2-weighted imaging, age, and serum biomarkers, the combined model allowed diagnosis of cirrhosis with moderate accuracy, independent of the size of focal liver lesions.

**Critical relevance statement:**

The fully automated convolutional neural network model utilizing pre-contrast MR imaging, age and serum biomarkers demonstrated moderate accuracy, outperforming FIB-4, APRI, and radiologists, independent of size of focal liver lesions, potentially facilitating noninvasive diagnosis of cirrhosis pending further validation.

**Key Points:**

- This fully automated convolutional neural network (CNN) model, using pre-contrast MRI, age, and serum biomarkers, diagnoses cirrhosis.
- The CNN model demonstrated an external testing dataset AUC of 0.86, independent of the size of focal liver lesions.
- The CNN model outperformed aminotransferase-to-platelet ratio index, fibrosis-4 index, and radiologists, potentially facilitating noninvasive diagnosis of cirrhosis.

**Graphical Abstract:**

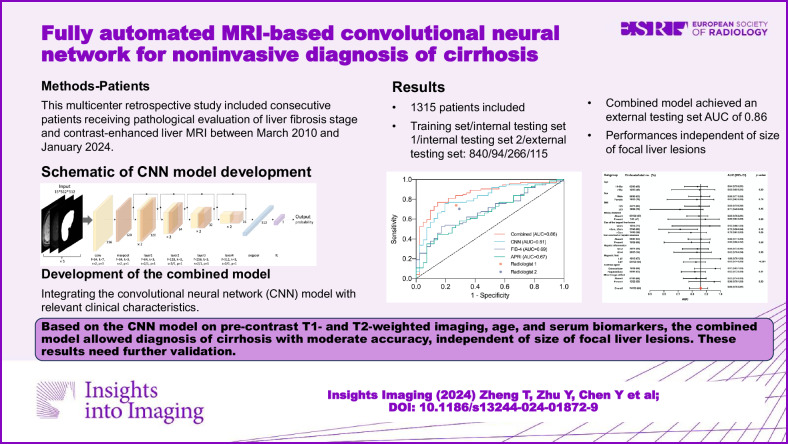

## Introduction

Cirrhosis is the end stage of liver fibrosis and results from chronic liver injury commonly caused by hepatitis B, hepatitis C, and alcohol [1]. Cirrhosis is the 11th leading cause of death globally [2] and represents the strongest risk factor for hepatocellular carcinoma (HCC) regardless of the etiology [3]. In patients with chronic liver disease, a diagnosis of cirrhosis not only affects prognosis, but also influences surveillance strategies, diagnosis, and suitability for surgical treatments of HCC [2, 4–6].

The gold standard for the diagnosis of cirrhosis is histopathological examination based on liver biopsy, resection, or transplantation. However, histopathological examination is prone to sampling bias [7, 8]. Furthermore, liver biopsy is invasive, while resection and transplantation cannot diagnose cirrhosis preoperatively. These drawbacks lead to the development of noninvasive diagnostic methods, with serum laboratory tests (e.g., fibrosis-4 index [FIB-4] and aminotransferase-to-platelet ratio index [APRI]) and liver stiffness measurement by ultrasound or MR elastography techniques being the most common [9, 10]. However, FIB-4 and APRI have insufficient diagnostic accuracy for cirrhosis (areas under the receiver operating characteristic curve [AUC], 0.65 to 0.74) [11, 12], and liver stiffness measurement is limited by a lack of consensus on cut-off values regarding different etiologies and vendors, confounded by the effects of several other non-fibrosis factors (e.g., liver inflammation, cholestasis), and requires dedicated hardware [13].

Routine MRI can depict the morphologic and functional changes of the liver and thus can also be used for cirrhosis diagnosis. MRI is particularly suitable for patients with suspected or confirmed malignancies for concurrent tumor detection, diagnosis, staging, aggressiveness evaluation and resectability assessment [14, 15]. However, the sensitivity (~ 70%) and reproducibility (moderate agreement) of manual assessment remained suboptimal based on MRI [16, 17]. In this context, two recent studies successfully applied convolutional neural network (CNN) to diagnose cirrhosis based on the hepatobiliary phase MR images, with AUCs reaching 0.84–0.85 [18, 19]. However, hepatobiliary contrast agents are less used in Western countries and may have degraded image quality in patients with severe liver dysfunction [20]. Furthermore, relevant serum biomarkers (e.g., markers of liver function and portal hypertension) that might potentially improve diagnostic accuracy were not analyzed in these models [13]. The effects of potential confounding factors (e.g., size of liver tumors) on liver morphological and texture alterations were not investigated.

Therefore, this study aimed to (1) develop and externally validate a fully annotated CNN model for diagnosis of cirrhosis based on liver MRI and serum biomarkers, (2) investigate the effects of potential confounders on model accuracies, and (3) compare the model performances with FIB-4, APRI and radiologists.

## Materials and methods

This multicenter, retrospective study was approved by the institutional review boards at four tertiary-care hospitals with waived informed consent.

### Patients

Between March 2010 and January 2024, consecutive adult patients who fulfilled the following inclusion criteria were identified: (1) adequate pathological evaluation of liver fibrosis through liver resection, transplantation, or biopsy; (2) underwent contrast-enhanced liver MRI within 3 months prior to surgery or around liver biopsy. Patients were excluded if they: (1) underwent any previous liver-directing treatment (e.g., liver resection and ablation) before MRI or between MRI and surgery/liver biopsy; (2) with inadequate MR image quality for assessment (e.g., severe MRI artifact); (3) with incomplete key lab test results (detailed below) within 3 months prior to surgery or liver biopsy. For patients with multiple qualified liver MRI, the one closest to surgery or liver biopsy was selected.

All patients included from center 1 between March 2010 and May 2021 were divided into a training dataset and an internal testing dataset 1 with a ratio of about 9:1 in a stratified manner to ensure a similar prevalence of cirrhosis between the two datasets. Patients included from center 1 between June 2021 and March 2023 were the internal testing dataset 2, while patients included from centers 2 to 4 were the external testing dataset.

Patient demographics, etiologies of the underlying liver diseases, type of histopathological specimen, and key laboratory results (e.g., aspartate aminotransferase [AST] and alanine aminotransferase [ALT]) within 3 months prior to surgery or liver biopsy were collected. FIB-4 and APRI were calculated as previously described [9, 10].

### Reference standard

Pathologic data retrieved from routine reports were used as the reference standard for the diagnosis of cirrhosis. In compliance with the institutional standard practice procedures, two liver pathologists who were aware of the clinical and imaging data reviewed all specimens in consensus. The liver fibrosis stage was determined according to grading and staging criteria for chronic hepatitis (Beijing, 1995) [21], which is modified from the Scheuer system, as follows: S0, no fibrosis; S1, enlargement and fibrosis of portal area; S2, formation of fibrous septum, but intact lobular structure; S3, fibrous septum with lobular structure distortion but no cirrhosis; and S4, early cirrhosis or definite cirrhosis. Fibrosis stage S4 was diagnosed as cirrhosis.

### MRI examination

The MR images were acquired on several 3.0-T or 1.5-T MR scanners (Supplementary Material 1 and Table S1). Liver MRI protocols included dual-echo images, T2-weighted images, and fat-suppressed T1-weighted dynamic phases.

### Imaging analysis

For the testing datasets, all MRIs were independently reviewed by two radiologists at center 1 with seven (radiologist 1) and three (radiologist 2) years of experience in liver MRI, respectively. The reviewers were blinded to the histopathologic, clinical and laboratory information. Each reviewer analyzed the MR images for the presence or absence of radiological cirrhosis, which was defined as unequivocal morphological alterations of the liver (e.g., surface nodularity, segmental volume redistribution, and parenchymal nodules), with or without manifestations of portal hypertension (portal-systemic collaterals, splenomegaly and/or ascites) [6, 22]. Radiologist 1 assessed image artifact (Supplementary Material 2) and presence of iron overload and liver steatosis.

Data on the number, size, location, and imaging diagnosis of focal liver lesions, bile duct dilatation and ascites were extracted from routine MRI report and verified by radiologist 1.

### Development of the CNN model

#### Image processing

Four major steps, including image co-registration, liver segmentation, image normalization, and augmentation were performed for image processing.

Specifically, the images of different sequences were first registered to the portal venous phase images using a self-developed rigid registration network (Supplementary Material 3 and Table S2). For liver segmentation, the outline of the liver was automatically extracted from each sequence using a self-developed segmentation framework to form the liver mask (Supplementary Material 4, Figs. S1 and S2, and Table S3), which helped the model to pay more attention to the liver area and reduce information redundancy of original images. Five slices were selected from the original MR images of each sequence, including the slice with the largest liver area and four slices located on the positions of 15 and 25% of the slice numbers above and below the maximum section within the liver region, and the corresponding liver mask of each slice was selected as input. Extreme values in individual pixels within the MR images were corrected by taking the 99.9% percentile of the image intensity range and clipping the pixels above this value. To keep the aspect ratio, each slice of the input was padded with a constant value of 0 to get the same width and height. Then the input was resized to 512 × 512 to provide rich image information for CNN model training. The interpolation methods adopted for original MR images and liver masks were linear interpolation and nearest neighbor interpolation, respectively. The input was finally normalized with min-max scaling. To reduce the potential bias caused by unbalanced data, the input was augmented in parallel online using rotation, zoom, contrast adjustment, Gaussian noise, and elastic distortion methods during the training process.

#### Development of the CNN model

Fivefold cross-validation strategy was applied to the training dataset during CNN training, and the averaged predicted probabilities served as the result (Supplementary Material 5).

Different combinations of pre-contrast T1- and T2-weighted imaging, and portal venous phase images were used to train separate CNN models, and the model with the optimal diagnostic performance for cirrhosis was selected for further analyses.

**Fig. 1:**
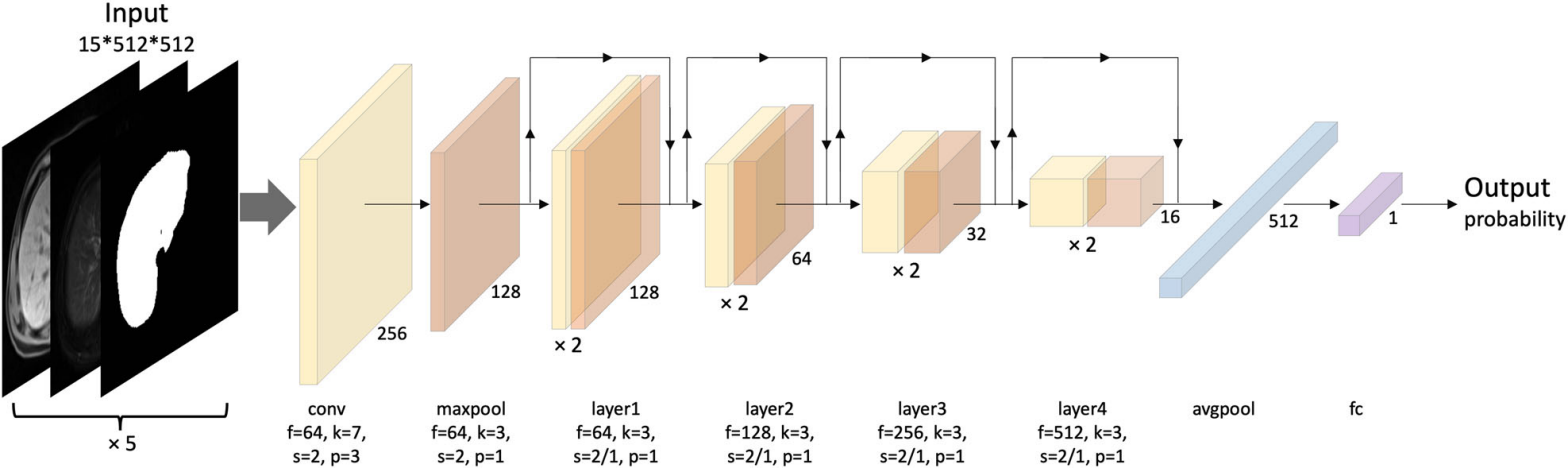
Schematic of convolutional neural network model development. avgpool, average pooling; conv, convolution; f, filter size; fc, full connected; k, kernel size; maxpool, max pooling; p, padding; s, stride

The CNN model was trained on a GeForce GTX 1080Ti (NVIDIA) graphic processing unit using Python 3.7 and PyTorch 1.7 backend. The architecture of the model is illustrated in Fig. 1 and detailed in Supplementary Material 5. ResNet-18 was applied as the model backbone [23]. The processed input was fed into the network, and the predicted probability was obtained. The network was trained using the Adam optimizer with an initial learning rate (LR) of 1e-3, and the LR was updated by ReduceLRonPlateau schedule. Weight decay was set to 1e-5. Binary cross-entropy loss was applied as loss function. Batch size was set to 32 and dropout rate was 0.3. For above hyperparameter optimization, manual search technique was used during CNN training. The network was trained for a maximum of 500 epochs or until the early stopping condition had been met, storing the network weights with the best validation AUC value.

#### CNN model visualization

Grad-CAM method was utilized to generate activation maps, which would indicate the areas that contributed most to the diagnosis of cirrhosis. We obtained Grad-CAM attributions using the pytorch-grad-cam library [24].

### Development of the combined model

Based on the training dataset, the combined model was established by integrating the CNN model with relevant clinical characteristics (Supplementary Material 6). Specifically, variance inflation factor (VIF) was first computed to detect multicollinearity. In cases of substantial collinearity (VIF > 5), univariable logistic regression analyses were performed to identify the variables with the strongest associations with cirrhosis (characterized by the largest absolute value of β coefficient). Afterwards, feature selection was performed among the remaining independent variables using the Select From Model algorithm combined with Random Forest estimator, where feature importance was computed using Gini importance (or mean decrease impurity). Finally, selected features were used to build the combined model using Random Forest. During training, grid search was used to determine the optimal hyperparameters of the estimator. Consistent with the CNN model, fivefold cross-validation method was also applied to the development of the combined model.

### Model evaluation

Based on the testing datasets, the combined model was validated and compared with the CNN model, FIB-4, APRI, and interpretations of two radiologists. Diagnostic performances were characterized by AUC, sensitivity, specificity, positive predictive value (PPV), negative predictive value (NPV), and accuracy.

### Statistical analysis

AUCs were compared using Delong test. PPVs and NPVs were compared using the weighted generalized score test proposed by Kosinski, while sensitivities, specificities, and accuracies were compared using McNemar’s test. Calibration plot was used to assess the agreement between the predicted risk and the actual risk. Decision curve analysis was conducted to calculate the clinical net benefit of the proposed models.

All statistical analyses were performed using SPSS software (version 26.0, IBM), Medcalc (version 20.100-64-bit) and R statistical software. Two-sided *p* < 0.05 was considered statistically significant.

## Results

### Patient characteristics

**Fig. 2:**
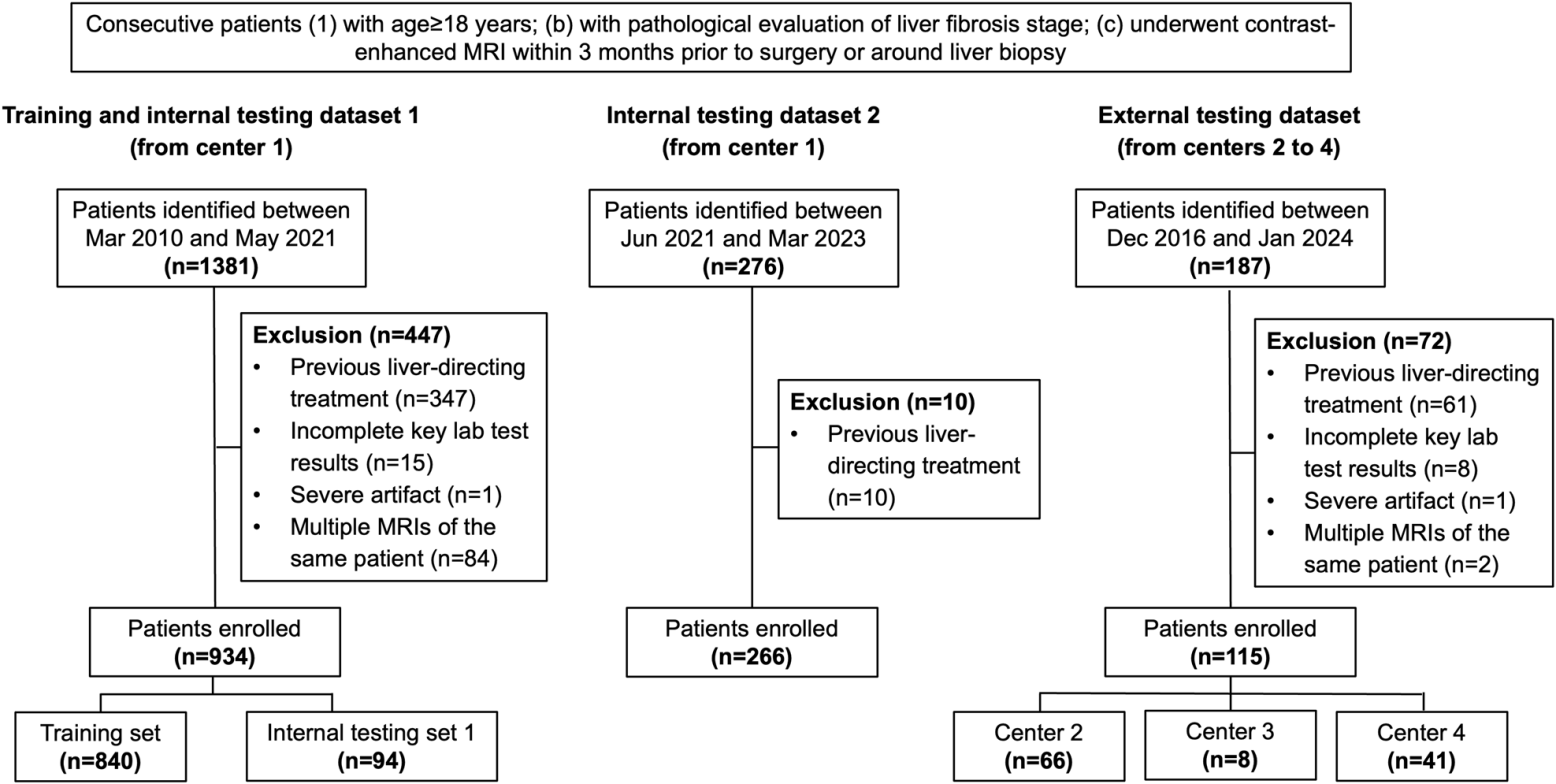
Flowchart of patient inclusion

A total of 1315 patients (median age, 54 years; interquartile range (IQR), 47–63 years; 1065 men) were included, 1200 (91%) (training dataset, *n* = 840; internal testing dataset 1, *n* = 94; internal testing dataset 2, *n* = 266) and 115 (9%) from center 1 and centers 2 to 4 (center 2, *n* = 66; center 3, *n* = 8; center 4, *n* = 41), respectively (Fig. 2, Table 1). Among them, 91% (1199/1315) of patients had chronic hepatitis B, and 65% (855/1315) had pathologically confirmed cirrhosis. Pathologic specimens were obtained by liver resection, transplantation, and biopsy in 94% (1231/1315), 2% (21/1315), and 5% (63/1315) of patients, respectively. The median time between liver MRI and surgery/biopsy was 9 days (IQR, 3–16 days).

**Table 1:** Demographic and clinical data of the total cohort, training dataset, and testing datasets

Characteristic	Total cohort *n* = 1315	Training dataset *n* = 840	Internal testing dataset 1		Internal testing dataset 2		External testing dataset	
			*n* = 94	*p*-value^f^	*n* = 266	*p*-value^f^	*n* = 115	*p*-value^f^
Age^a^	54 (47, 63)	53 (46, 63)	54 (47, 62)	0.79	57 (49, 66)	**< 0.001**	53 (48, 58)	0.55
Sex				0.30		0.32		0.60
Male	1065 (81.0)	689 (82.0)	73 (77.7)		211 (79.3)		92 (80.0)	
Female	250 (19.0)	151 (18.0)	21 (22.3)		55 (20.7)		23 (20.0)	
Etiology of liver disease^b^								
HBV	1199 (91.2)	804 (95.7)	90 (95.7)	1.00	206 (77.4)	**< 0.001**	99 (86.1)	**< 0.001**
HCV	33 (2.5)	15 (1.8)	5 (5.3)	0.06	10 (3.8)	0.06	3 (2.6)	0.81
Alcohol	11 (0.8)	5 (0.6)	2 (2.1)	0.15	3 (1.1)	0.63	1 (0.9)	0.54
Others or unknown^c^	98 (7.5)	36 (4.3)	2 (2.1)	0.47	48 (18.0)	**< 0.001**	12 (10.4)	**0.005**
Child-Pugh class^d^				**0.001**		**0.006**		0.133
A	1229 (93.5)	800 (95.2)	85 (90.4)		240 (90.2)		104 (90.4)	
B	77 (5.9)	38 (4.5)	6 (6.4)		23 (8.6)		10 (8.7)	
C	8 (0.6)	2 (0.2)	3 (3.2)		3 (1.1)		0 (0.0)	
MELD score^a^	7 (7, 8)	7 (6, 8)	7 (7, 9)	0.51	7 (7, 8)	0.11	8 (7, 9)	**0.04**
ALBI grade				**0.004**		0.26		**< 0.001**
1	968 (73.6)	634 (75.5)	64 (68.1)		208 (78.2)		62 (53.9)	
2	332 (25.2)	200 (23.8)	26 (27.7)		54 (20.3)		52 (45.2)	
3	15 (1.1)	6 (0.7)	4 (4.3)		4 (1.5)		1 (0.9)	
BMI^a,d^	23.4 (21.4, 25.4)	23.2 (21.3, 25.5)	23.3 (21.1, 25.3)	0.95	23.6 (22.0, 25.4)	0.17	23.5 (21.3, 25.0)	0.64
Pathology specimen				**0.03**		**0.049**		**< 0.001**
Biopsy	63 (4.8)	34 (4.0)	2 (2.1)		7 (2.6)		20 (17.4)	
Resection	1231 (93.6)	797 (94.9)	88 (93.6)		251 (94.4)		95 (82.6)	
Transplantation	21 (1.6)	9 (1.1)	4 (4.3)		8 (3.0)		0 (0.0)	
Liver fibrosis stage				0.11		**< 0.001**		**0.04**
S0	34 (2.6)	29 (3.5)	4 (4.3)		1 (0.4)		0 (0.0)	
S1	60 (4.6)	27 (3.2)	6 (6.4)		18 (6.8)		9 (7.8)	
S2	187 (14.2)	121 (14.4)	7 (7.4)		45 (16.9)		14 (12.2)	
S3	179 (13.6)	129 (15.4)	10 (10.6)		22 (8.3)		18 (15.7)	
S4	855 (65.0)	534 (63.6)	67 (71.3)		180 (67.7)		74 (64.3)	
Liver lesions^b^								
None	104 (7.9)	60 (7.1)	9 (9.6)	0.39	20 (7.5)	0.84	15 (13.0)	**0.03**
HCC	1056 (80.3)	676 (80.5)	75 (79.8)	0.87	212 (8.0)	0.78	93 (80.9)	0.92
ICC	75 (5.7)	45 (5.4)	2 (2.1)	0.27	24 (9.0)	**0.03**	4 (3.5)	0.39
cHCC-CCA	22 (1.7)	11 (1.3)	3 (3.2)	0.33	7 (2.6)	0.23	1 (0.9)	1.00
Metastases	12 (0.9)	10 (1.2)	0 (0.0)	0.59	2 (0.8)	0.79	0 (0.0)	0.49
Perihilar cholangiocarcinoma/carcinoma of the gall bladder affecting the liver	19 (1.4)	18 (2.1)	1 (1.1)	0.75	0 (0.0)	**0.03**	0 (0.0)	0.22
Others^e^	44 (3.3)	32 (3.8)	5 (5.3)	0.67	4 (1.5)	0.07	3 (2.6)	0.71
Size of the largest liver lesion^a,d^	3.5 (2.3, 5.5)	3.5 (2.4, 5.7)	3.0 (2.0, 5.9)	0.20	3.0 (2.1, 5.0)	**0.008**	4.0 (2.5, 6.6)	**0.03**
Magnetic field				0.16		**< 0.001**		**< 0.001**
1.5 T	652 (49.6)	519 (61.8)	51 (54.3)		67 (25.2)		15 (13.0)	
3.0 T	663 (50.4)	321 (38.2)	43 (45.7)		199 (74.8)		100 (87.0)	
Contrast agent				0.21		**< 0.001**		**< 0.001**
Extracellular	812 (61.7)	622 (74.0)	64 (68.1)		98 (36.8)		28 (24.3)	
Hepatobiliary	503 (38.3)	218 (26.0)	30 (31.9)		168 (63.2)		87 (75.7)	

Unless otherwise specified, data are number of patients, with percentages in parentheses. The bold values are significantly different between the training and testing datasets. For example, the percentage of HBV etiology was higher in the training dataset compared to internal testing dataset 1 (95.7% vs. 77.4%, *p* < 0.001)

*ALBI* albumin-bilirubin, *BMI* body mass index, *cHCC-CCA* combined hepatocellular-cholangiocarcinoma, *HBV* hepatitis B virus, *HCC* hepatocellular carcinoma

^a^ Data are median with interquartile range in parentheses

^b^ More than one etiology or liver lesion could be present in each patient. Liver lesions do not include cyst, cavernous hemangioma, or regenerative nodule

^c^ Others include autoimmune liver disease, non-alcoholic steatohepatitis, and drug-induced liver injury

^d^ Data presented from patients who have complete data of Child-Pugh class, BMI or who have at least one liver lesion

^e^ Others include other neoplasms, premalignant lesion, focal nodular hyperplasia, inflammatory pseudotumor, abscess, and parasite infection

^f^ *p*-values were computed by comparing the training and testing sets with the use of the Mann–Whitney U test for continuous variables (i.e., age, MELD score, BMI, and size of the largest liver lesion) and either the χ2 test or Fisher’s exact test, where applicable, for categorical variables (e.g., sex and etiology of liver disease)

Regarding focal liver lesions, 8% (104/1315) of patients had no intrahepatic lesions, 80% (1056/1315) had HCC, and 13% (172/1315) had other kinds of liver lesions, excluding simple cysts, cavernous hemangiomas, or regenerative nodules. Solitary and multiple liver lesions were observed in 77% (1014/1315) and 15% (197/1315) of patients, respectively. The median size of the largest liver lesion was 3.5 cm (IQR, 2.3–5.5 cm).

The proportion of pathological cirrhosis was consistent between the training and testing datasets (all *p* < 0.05). Child-Pugh class (*p* = 0.001), albumin-bilirubin grade (*p* = 0.004), and source of pathology specimen (*p* = 0.03) were different between the training dataset and internal testing dataset 1. Compared with the training dataset, the internal testing dataset 2 and external testing dataset patients had less frequent HBV infection (both *p* < 0.001), smaller (internal testing set 2) or larger (external testing dataset) liver lesions (*p* = 0.008 and *p* = 0.03), more frequent 3.0-T MRI (both *p* < 0.001) and hepatobiliary contrast agent-enhanced MRI (both *p* < 0.001).

### Development of the CNN and combined models

The final CNN model was constructed based on pre-contrast T1- and T2-weighted imaging due to optimal training dataset AUC and balanced sensitivity and specificity among all sequence combinations (Table S4).

For the combined model, no multicollinearity was found among variables (Table S5), and the CNN model-based cirrhosis probability, age, platelet count, total bilirubin, albumin, AST, ALT, alkaline phosphatase (ALP), gamma-glutamyl transferase (GGT), and international normalized ratio were selected to build the combined model. CNN model-based cirrhosis probability and platelet count had the highest importance score in all folds, which ranged from 0.32–0.60 and 0.10–0.15, respectively (Table S6).

Figure S4 shows the receiver operating characteristic (ROC) curves of the CNN and combined models on the training dataset.

### Comparing the performance between the models

**Fig. 3:**
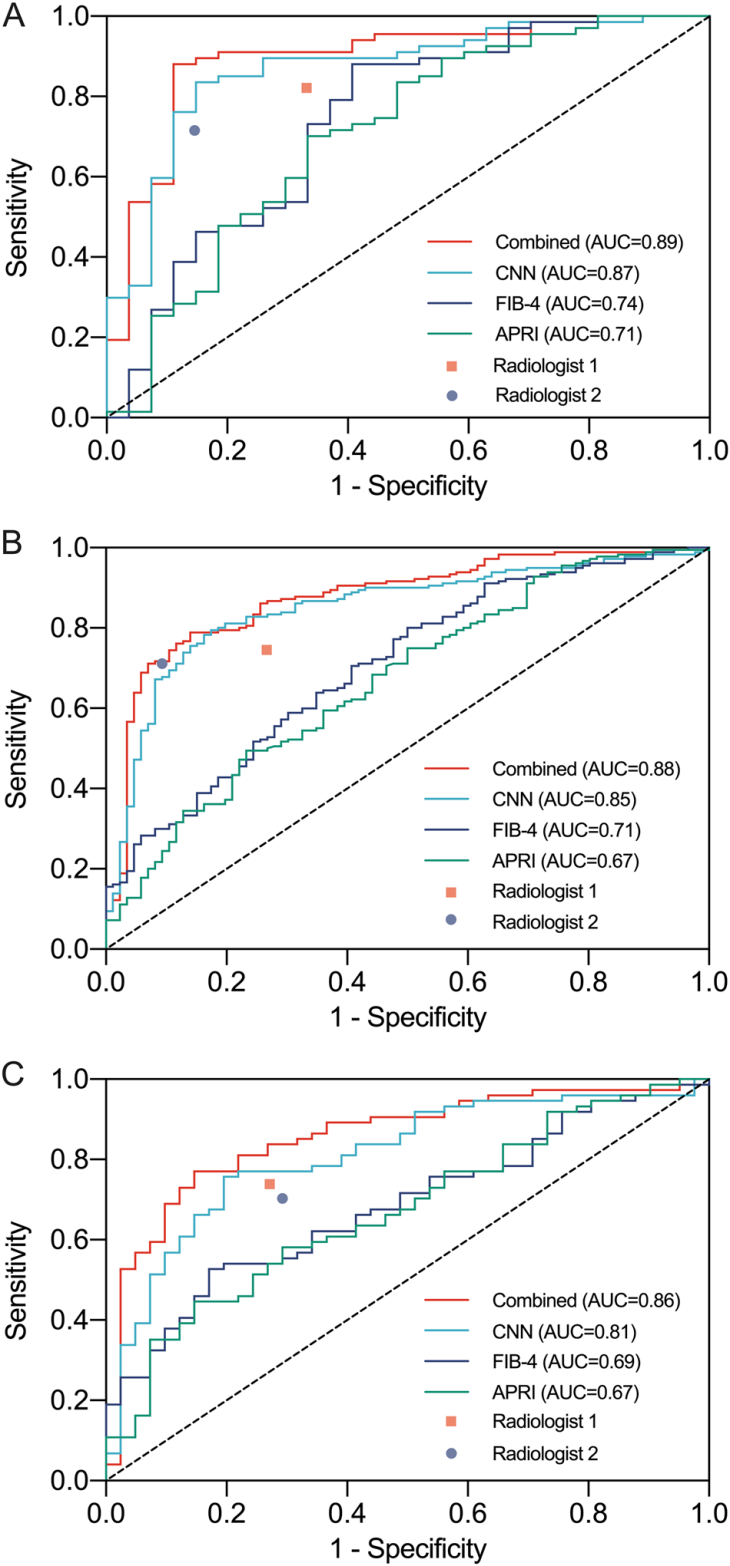
Receiver operating characteristic curves for diagnosis of cirrhosis on the internal testing dataset 1 (**A**), internal testing dataset 2 (**B**), and external testing dataset (**C**). The area under the receiver operating characteristic curve of the combined model was higher than those of FIB-4, APRI and radiologists. APRI, aminotransferase-to-platelet ratio index; AUC, area under the receiver operating characteristic curve; CNN, convolutional neural network; FIB-4, fibrosis-4 index

Table 2 summarizes the diagnostic performance of different models and two radiologists for cirrhosis on the testing datasets and the ROC curves are shown in Fig. 3.

**Table 2:** Comparison of diagnostic performance of the combined model, CNN model, FIB-4, APRI, and radiologists for cirrhosis on the internal and external testing datasets

	Combined model	CNN model	FIB-4^a^	APRI^a^	Radiologist 1	Radiologist 2
Internal testing dataset 1						
Cut-off^b^	> 0.53405	> 0.54343	> 2.26352	> 0.54094	/	/
AUC	0.89 (0.81–0.95)	0.87 (0.78–0.93)	0.74 (0.64–0.82)	0.71 (0.61–0.80)	0.74 (0.64–0.83)	0.78 (0.69–0.86)
*p*-value^c^	/	0.36	0.008	0.003	0.006	0.04
Sensitivity	90% (80%–96%)	87% (76%–94%)	69% (56%–79%)	72% (59%–82%)	82% (71%–90%)	72% (59%–82%)
*p*-value^c^	/	0.50	0.001	0.02	0.23	0.004
Specificity	81% (62%–94%)	74% (54%–89%)	67% (46%–83%)	63% (42%–81%)	67% (46%–83%)	85% (66%–96%)
*p*-value^c^	/	0.50	0.29	0.18	0.34	1.00
PPV	92% (84%–96%)	89% (81%–94%)	84% (75%–90%)	83% (74%–89%)	86% (78%–91%)	92% (83%–97%)
*p*-value^c^	/	0.12	0.04	0.03	0.15	1.00
NPV	76% (60%–87%)	69% (54%–81%)	46% (35%–57%)	47% (36%–59%)	60% (46%–73%)	55% (45%–65%)
*p*-value^c^	/	0.07	< 0.001	0.002	0.06	0.006
Accuracy	87% (79%–93%)	83% (74%–90%)	68% (58%–77%)	69% (59%–78%)	78% (68%–86%)	76% (66%–84%)
*p*-value^c^	/	0.13	< 0.001	0.003	0.08	0.03
True positive	60	58	46	48	55	48
False positive	5	7	9	10	9	4
False negative	7	9	21	19	12	19
True negative	22	20	18	17	18	23
Internal testing dataset 2						
Cut-off^b^	> 0.53405	> 0.54343	> 2.26352	> 0.54094	/	/
AUC	0.88 (0.83–0.91)	0.85 (0.80–0.89)	0.71 (0.65–0.76)	0.67 (0.61–0.73)	0.74 (0.68–0.79)	0.81 (0.76–0.85)
*p*-value^c^	/	0.03	< 0.001	< 0.001	< 0.001	0.01
Sensitivity	87% (81%–91%)	81% (75%–87%)	64% (56%–71%)	59% (52%–67%)	74% (67%–81%)	71% (64%–78%)
*p*-value^c^	/	0.01	< 0.001	< 0.001	0.002	< 0.001
Specificity	71% (60%–80%)	79% (69%–87%)	64% (53%–74%)	62% (51%–72%)	73% (63%–82%)	91% (82%–96%)
*p*-value^c^	/	0.09	0.36	0.18	0.86	0.002
PPV	86% (82%–90%)	89% (84%–92%)	79% (73%–83%)	76% (71%–81%)	85% (80%–89%)	94% (89%–97%)
*p*-value^c^	/	0.13	0.01	0.001	0.78	0.008
NPV	72% (63%–79%)	67% (59%–73%)	46% (40%–52%)	42% (36%–48%)	58% (51%–64%)	60% (54%–66%)
*p*-value^c^	/	0.09	< 0.001	< 0.001	0.006	0.02
Accuracy	82% (76%–86%)	80% (75%–85%)	64% (58%–70%)	60% (54%–66%)	74% (68%–79%)	77% (72%–82%)
*p*-value^c^	/	0.70	< 0.001	< 0.001	0.03	0.27
True positive	156	146	115	107	134	128
False positive	25	18	31	33	23	8
False negative	24	34	65	73	46	52
True negative	61	68	55	53	63	78
External testing dataset						
Cut-off^b^	> 0.58556	> 0.57295	> 3.03248	> 1.03125	/	/
AUC	0.86 (0.78–0.91)	0.81 (0.73–0.88)	0.69 (0.59–0.77)	0.67 (0.58–0.76)	0.73 (0.64–0.81)	0.71 (0.61–0.79)
*p*-value^c^	/	0.02	0.001	< 0.001	0.02	0.006
Sensitivity	84% (73%–91%)	77% (66%–86%)	62% (50%–80%)	65% (53%–76%)	73% (61%–83%)	70% (59%–80%)
*p*-value^c^	/	0.13	0.003	0.007	0.10	0.05
Specificity	73% (57%–86%)	68% (52%–82%)	66% (49%–80%)	54% (37%–69%)	73% (57%–86%)	71% (54%–84%)
*p*-value^c^	/	0.73	0.58	0.08	1.00	1.00
PPV	85% (77%–90%)	81% (73%–87%)	77% (67%–84%)	72% (64%–79%)	83% (74%–89%)	81% (72%–88%)
*p*-value^c^	/	0.30	0.08	0.005	0.67	0.48
NPV	71% (59%–81%)	62% (51%–72%)	49% (40%–58%)	46% (36%–56%)	60% (50%–69%)	57% (47%–66%)
*p*-value^c^	/	0.045	0.001	< 0.001	0.08	0.04
Accuracy	80% (72%–87%)	74% (65%–82%)	63% (54%–72%)	61% (51%–70%)	73% (64%–81%)	70% (61%–79%)
*p*-value^c^	/	0.12	0.003	< 0.001	0.20	0.11
True positive	62	57	46	48	54	52
False positive	11	13	14	19	11	12
False negative	12	17	28	26	20	22
True negative	30	28	27	22	30	29

Data in parentheses are 95% confidence interval

*APRI* aminotransferase-to-platelet ratio index, *AUC* area under the receiver operating characteristic curve, *CNN* convolutional neural network, *FIB-4* fibrosis-4 index, *NPV* negative predictive value, *PPV* positive predictive value

^a^ The calculation formulas were as follows: FIB-4 = (age [year] × AST [U/L]) / (platelet count [10^9^/L] × (ALT [U/L])^1/2^); APRI = (AST (/upper limit of normal) / platelet count [10^9^/L]) × 100 [9, 10]

^b^ Cut-off values were selected based on the receiver operating characteristic and Youden index in the training dataset. Cut-offs of combined and CNN models represent the model outputs for the combined model and the CNN model

^c^ *p*-values were calculated in comparison to the combined model. AUCs were compared using Delong test. PPVs and NPVs were compared using the weighted generalized score test proposed by Kosinski, while sensitivities, specificities, and accuracies were compared using McNemar’s test

Briefly, the combined model achieved an external testing dataset AUC of 0.86 (95% confidence interval [CI], 0.78–0.91) for diagnosing cirrhosis, which was higher than the CNN model (AUC: 0.81, 95% CI: 0.73–0.88, *p* = 0.02), FIB-4 (AUC: 0.69, 95% CI: 0.59–0.77, *p* = 0.001), APRI (AUC: 0.67, 95% CI: 0.58–0.76, *p* < 0.001) and two radiologists (radiologist 1 AUC: 0.73, 95% CI: 0.64–0.81, *p* = 0.02; radiologist 2 AUC: 0.71, 95% CI: 0.61–0.79, *p* = 0.006).

The sensitivity, specificity, PPV, NPV, and accuracy of the combined model on the external testing dataset were 84%, 73%, 85%, 71%, and 80%, respectively. The sensitivity of the combined model was higher than FIB-4 (62%, *p* = 0.003) and APRI (65%, *p* = 0.007).

As shown in calibration plots, the combined model revealed better consistency between the predicted decision and actual diagnosis than FIB-4 and APRI (Fig. S5A, C, E). Decision curves showed that the prognostic score of the combined model provided good net benefit across the reasonable range of threshold probabilities (Fig. S5B, D, F).

**Fig. 4:**
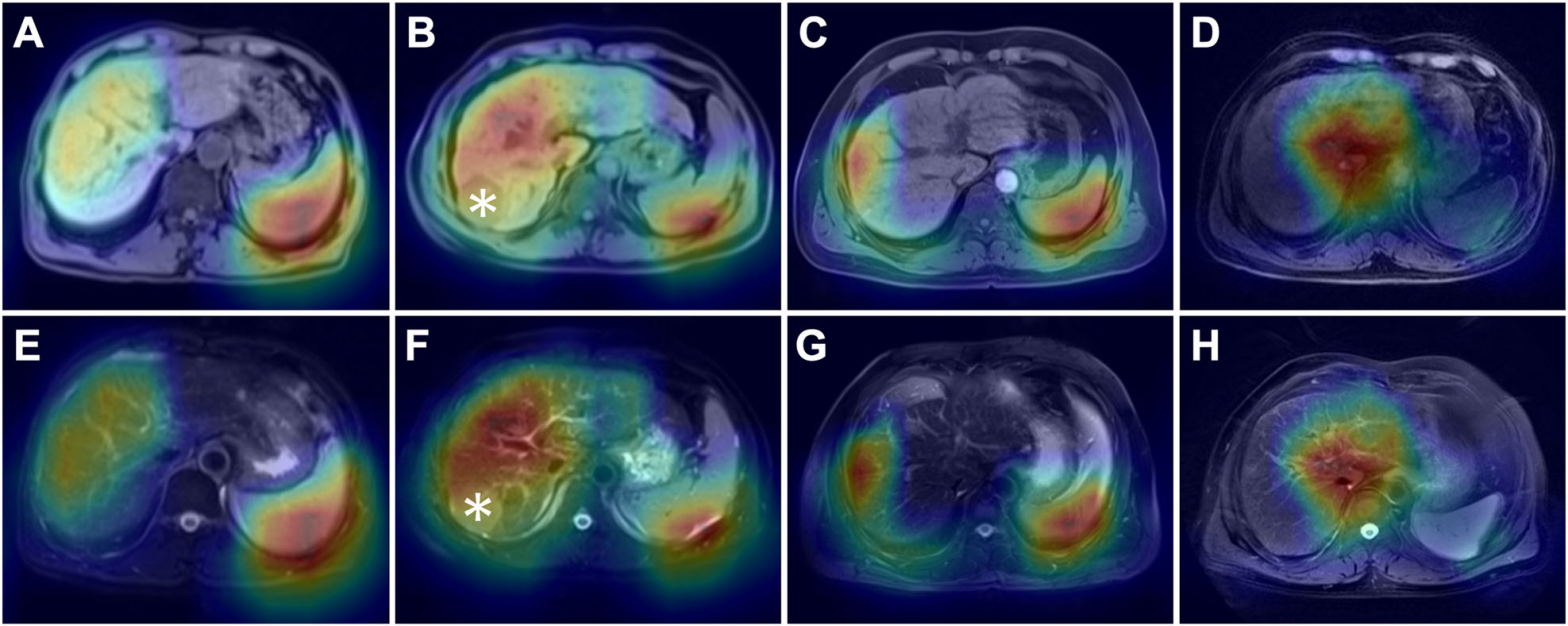
Representative activation maps overlaid on pre-contrast T1-weighted (**A**–**D**) and T2-weighted (**E**–**H**) axial images in patients with cirrhosis who were correctly classified by the CNN model. The liver parenchyma, liver surface, portal hepatis, and spleen are highlighted in these maps, which indicates that information from these areas contributes to the prediction of cirrhosis. Note the hepatocellular carcinoma lesions in the liver (**B**, **F**, asterisk). CNN, convolutional neural network

Representative activation maps of patients with cirrhosis who were correctly classified by the CNN model indicated that liver parenchyma, liver surface, portal hepatis, and spleen contributed most to the diagnostic decisions (Fig. 4).

**Fig. 5:**
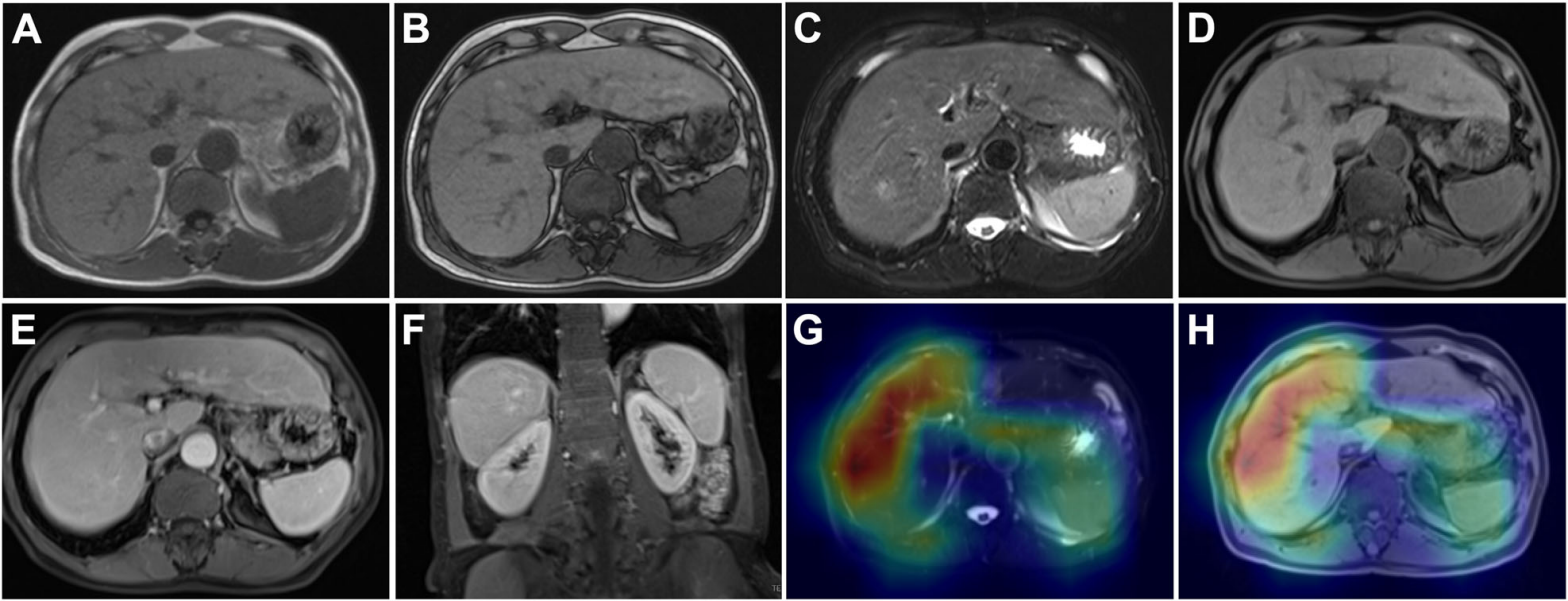
Extracellular contrast agent-enhanced MRI of a 67-year-old male patient with cirrhosis and chronic hepatitis B. In- and opposed-phase images (**A**, **B**), T2-weighted image (**C**), pre-contrast T1-weighted image (**D**), and portal venous phase images (**E**, **F**) revealed no surface nodularity or other morphological changes and no manifestations of portal hypertension. Activation maps overlaid on T2-weighted (**G**) and pre-contrast T1-weighted (**H**) images showed highlights in liver parenchyma, liver surface, and spleen. The combined model- and CNN model-based cirrhosis probability were 0.77 and 0.64, respectively, which both lead to a diagnosis of cirrhosis. Neither radiologist diagnosed this case as cirrhosis

Figure 5 shows the MR images of a patient with cirrhosis who was correctly diagnosed by the combined and CNN models but misdiagnosed by both radiologists.

### Subgroup analyses of the combined model

**Fig. 6:**
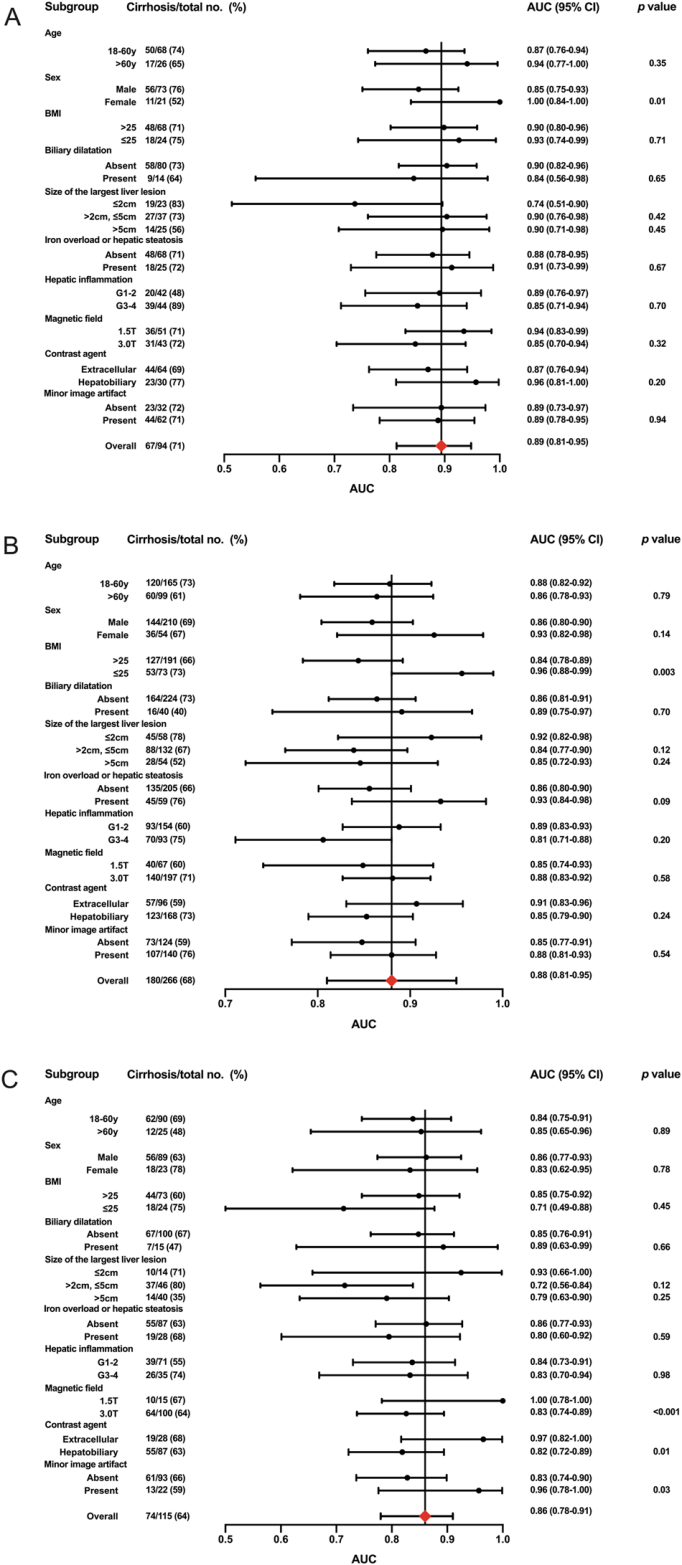
Forest plot showing the AUC, 95% confidence interval, and *p*-value of the combined model for diagnosis of cirrhosis in subgroups of the internal testing dataset 1 (**A**), internal testing dataset 2 (**B**), and external testing dataset (**C**). AUC, the area under the receiver operating characteristic curve; BMI, body mass index; CI, confidence interval

On the internal testing dataset 1, the AUC of the combined model was higher in the female group than in the male group (1.00 vs. 0.85, *p* = 0.01) (Fig. 6A). On the internal testing dataset 2, the AUC of the combined model was higher in the BMI ≤ 25 group than in the BMI > 25 group (0.96 vs. 0.84, *p* = 0.003) (Fig. 6B). On the external testing dataset, the AUC of the combined model was higher in the 1.5-T MRI group (1.00 vs. 0.83, *p* < 0.001), extracellular contrast agent group (0.97 vs. 0.82, *p* = 0.01) and minor image artifact group (0.96 vs. 0.83, *p* = 0.03) (Fig. 6C).

There was no significant difference in the AUCs of the combined model among subgroups with different size of the largest liver lesion (≤ 2 cm vs. > 2 cm, ≤ 5 cm, all *p* > 0.05; ≤ 2 cm vs. > 5 cm, all *p* > 0.05).

## Discussion

Noninvasive diagnosis of cirrhosis is critical for patient management but challenging with conventional MRI. In this study, we developed and externally validated a convolutional neural network-based model for fully automated diagnosis of cirrhosis by integrating liver MRI and relevant clinical characteristics. The combined model based on pre-contrast T1- and T2-weighted imaging, age, and eight serum biomarkers allowed diagnosis of cirrhosis with an external testing dataset area under the receiver operating characteristic curve (AUC) of 0.86, independent of other radiological and clinical factors that might impact the diagnostic performances of the model, and outperformed fibrosis-4 index (AUC: 0.69, *p* = 0.001), aminotransferase-to-platelet ratio index (AUC: 0.67, *p* < 0.001), and two radiologists (AUC: 0.73, *p* = 0.02; AUC: 0.71, *p* = 0.006).

While most existing diagnostic techniques for cirrhosis required manual annotations during modeling [18, 25, 26], the CNN model developed in this work was based on fully automated segmentations of the liver. This approach may provide a more easily applicable and reproducible diagnostic tool for cirrhosis. Interestingly, the CNN model based on pre-contrast T1- and T2-weighted imaging demonstrated the optimal diagnostic performance among all sequence combinations. Despite our initial efforts in investigating as many imaging sequences (including post-contrast images) as possible during modeling to maximize the diagnostic performance, the information on cirrhosis seemed to have been well-represented by pre-contrast images, which might be due to complementary image information of T1- and T2-weighted imaging and limited incremental value of vascular changes added by portal venous phase images. These results were clinically relevant, as our model may be extrapolated to a wider range of clinical applications without the need of contrast agents.

Recently, Yosaka et al [18] and Hectors et al [19] reported on CNN systems for diagnosis of cirrhosis using hepatobiliary phase MR images with AUCs of 0.84 and 0.85, which were comparable to that of our CNN model (AUC: 0.81). Nowak et al [26] developed a deep transfer learning system for detecting cirrhosis based on T2-weighted imaging, which achieved an almost perfect AUC of 0.99 for the testing dataset. However, in their study, only patients with histologically or clinically defined liver cirrhosis and those with healthy livers were enrolled, which may limit model generalizability.

Serum biomarkers were incorporated into the combined model in this study. These biochemical markers reflect liver synthetic function (albumin), damage and inflammation (AST, ALT and GGT), cholestasis (total bilirubin, ALP and GGT), and portal hypertension (platelet count) [13], and thus may facilitate the diagnosis of cirrhosis. In fact, AST, ALT, albumin and platelet count have been incorporated into several established scores for noninvasive assessment of liver fibrosis (e.g., FIB-4, APRI, and non-alcoholic fatty liver disease fibrosis score (NFS)) [11, 13]. The combined model outperformed the widely used FIB-4 and APRI and two radiologists, which highlights the incremental values of the combined model to supplement existing systems for noninvasive diagnosis of cirrhosis.

The combined model developed in this study was robust in variable clinical settings. In particular, over 90% of patients enrolled in our study had focal liver lesions, mainly HCC (80%). Subgroup analyses indicated that the diagnostic performance of the combined model was independent of the size of focal liver lesions, which implies its potential clinical application to facilitate surgical treatment decision in patients with suspected or confirmed liver cancer. However, there were significant differences in the diagnostic performance across subgroups with different sex, BMI, magnetic field, contrast agent, presence or absence with minor artifact. These differences may indicate better or worse performance in specific subgroups but may also result from underpowered subgroup analyses due to small or unbalanced sample sizes. Future studies are required to validate these differences, particularly in small subgroups (e.g., female group and BMI ≤ 25 group) that tend to be under-presented. Furthermore, the use of different manufacturers (GE vs. uMR vs. Philips vs. Siemens) could potentially influence the diagnostic performance of our model. However, the data from our study do not support making meaningful comparisons between manufacturers in subgroup analyses. Platelet count decrease due to other reasons (e.g., non-cirrhotic portal hypertension) might contribute to false positives, while no significant imaging feature and normal lab test results might lead to false negatives (Table S7).

Noteworthily, the expandability of deep learning is critical for confident clinical adoptions. Despite the preliminary efforts to reveal the important regions focused by the CNN model on the activation maps, the details of how the CNN model could perform better than radiologists are still unclear. Therefore, future studies are encouraged to further unveil the “black box” of deep learning for cirrhosis diagnosis.

This study had several limitations. First, as a retrospective study, potential selection bias may exist and impact our results. Particularly, over 90% of the included patients had an etiology of hepatitis B virus infection and Child-Pugh class A and pediatric patients were not included in our study. This may limit the extrapolations to patients with other etiologies or worse liver function and pediatric patients. Second, the Beijing criteria for liver fibrosis staging was employed as the reference standard, which is not frequently used in Western practice. Third, the diagnostic AUC achieved by the combined model was moderate (below 0.90), which may be insufficient for establishing a diagnosis of cirrhosis in clinical practice on its own. In future research, we will continuously optimize the model by exploring additional strategies, including refining the input variables, expanding the dataset, and employing advanced modeling techniques. Fourth, although subgroup analyses revealed robust diagnostic performance across patients with different sizes of the largest liver lesions, the selected input slices may not cover all the liver lesions. Therefore, further studies are required to evaluate model utility in patients with liver lesions. Fifth, although our model achieved similar diagnostic performance with MR elastography as reported in recent meta-analyses (AUC: 0.92 to 0.93) [27, 28], our retrospective design lacked sufficient data for a head-to-head comparison because MR elastography is not routinely used in our medical centers. Future prospective study is needed to evaluate our combined model against MR elastography. However, despite the high accuracy of MR elastography in diagnosing cirrhosis, it may be less available at different centers. Sixth, the model performance compared with a combination of radiologists and serum biomarkers and the model performance on a healthy population is unknown. Further validation is needed with prospective cohort. Finally, although each step was fully automated, we have not integrated it into a single pipeline. We acknowledge that this may necessitate some manual input within the process. Developing a software is the future direction of our research.

In conclusion, based on 1315 patients with pathological evaluation of liver fibrosis stage who underwent contrast-enhanced liver MRI within 3 months, we developed and externally validated a combined model integrating pre-contrast T1- and T2-weighted imaging, age, and eight serum biomarkers. The model allowed diagnosis of cirrhosis with moderate accuracy, independent of size of focal liver lesions, and outperformed FIB-4, APRI and radiologists.

## Supplementary information

ELECTRONIC SUPPLEMENTARY MATERIAL

## Data Availability

The datasets generated or analyzed during the study are available from the corresponding author upon reasonable request.

## Abbreviations

ALP: Alkaline phosphatase
ALT: Alanine aminotransferase
APRI: Aminotransferase-to-platelet ratio index
AST: Aspartate aminotransferase
AUC: Areas under the receiver operating characteristic curve
CI: Confidence interval
CNN: Convolutional neural network
FIB-4: Fibrosis-4 index
GGT: Gamma-glutamyl transferase
HCC: Hepatocellular carcinoma
IQR: Interquartile range
NPV: Negative predictive value
PPV: Positive predictive value
ROC: Receiver operating characteristic
VIF: Variance inflation factor

## Code availability

The underlying code for this study performing the analyses on the training/testing datasets is not publicly available but may be made available to qualified researchers on reasonable request from the corresponding author.
