# Supplementary material for: Fully automated MRI-based convolutional neural network for noninvasive diagnosis of cirrhosis
Source: Insights Imaging. 2024 Dec 12;15:298. doi: 10.1186/s13244-024-01872-9 (PMC11638457; doi:10.1186/s13244-024-01872-9)
Supplement: Supplementary file 1 — ELECTRONIC SUPPLEMENTARY MATERIAL [file 13244_2024_1872_MOESM1_ESM.pdf]

Fully automated MRI-based convolutional neural network for noninvasive diagnosis of cirrhosis

ELECTRONIC SUPPLEMENTARY MATERIAL

Contents

Supplementary Material 1: MRI acquisition protocols 2

Table S1 MRI sequences and parameters 4

Supplementary Material 2: Assessment of image artifact 8

Supplementary Material 3: Image co-registration 9

Table S2 The Dice and ASD results of 3D image registration network 9

Supplementary Material 4: Liver segmentation 10

Figure S1: The one-to-multiple unsupervised domain adaptation (OMUDA) framework 11

Figure S2: Segmentation results of different methods on the internal testing set 12

Table S3 Quantitative liver segmentation results of OMUDA framework on the internal testing set 13

Supplementary Material 5: CNN model training 14

Figure S3 Loss curves of derivation and validation sets of five-fold cross-validation experiments 15

Supplementary Material 6: Development of the combined model 16

Table S4 Performance of CNN models with different combinations of MRI sequences on the training set 17

Table S5 Multicollinearity test based on variance inflation factor 18

Table S6 Importance score of variables in the combined model 19

Figure S4 Receiver operating characteristic curves for diagnosis of cirrhosis on the training set 20

Figure S5 Calibration curves and decision curves for diagnosis of cirrhosis on the testing sets 21

Table S7 Examples of possible reasons for false positive and false negative cases of the combined model on the testing sets 22

Supplementary Material 1: MRI acquisition protocols

Training and internal testing center

All contrast-enhanced MR examinations were performed with one of the following eight types of 3.0 T or 1.5 T MR scanners (Siemens MAGNETOM Skyra; Siemens Avanto; Siemens TrioTim; GE Discovery 750w; GE SIGNA™ Premier; GE SIGNA™ Architect; Phillips Ingenia Elition X; uMR588) using either extracellular contrast agent (ECA-MRI) or gadoxetate disodium (EOB-MRI). All patients were asked to fast for 6-8 hours prior to MR examinations. The liver MRI protocols included (1) in- and opposed-phase T1-weighted imaging; (2) T2-weighted imaging; (3) diffusion-weighted imaging (b values: 0, 50, 500, 800, 1000, and 1200 s/mm^2 [Siemens MAGNETOM Skyra]; 0, 50, 800 s/mm^2 [Siemens Avanto]; 0, 50, 600 s/mm^2 [Siemens TrioTim]; 0, 50, 1000 s/mm^2 [Siemens Avanto; GE SIGNA™ Premier]; 50, 800, 1000, and 1200 s/mm^2 [GE Discovery 750w]; 0, 50, and 1000 s/mm^2 [GE SIGNA™ Architect]; 0, 200, 1000 s/mm^2 [Phillips Ingenia Elition X]; 0, 50, 1000, 1500 s/mm^2 [uMR588]) with apparent diffusion coefficient (ADC) maps reconstructed using the mono-exponential model; and (4) fat-suppressed T1-weighted dynamic phases including the pre-contrast phase, late arterial phase, portal venous phase (60s after the start of contrast media injection), delayed phase (for ECA-MRI, 180s after the start of contrast media injection) or transitional phase (for EOB-MRI, 5min after the start of contrast media injection), and hepatobiliary phase (for EOB-MRI, 20min after the start of contrast media injection). The arterial phase images were acquired with either a bolus-tracking method (acquisition triggered 7s after the arrival of the contrast bolus in the celiac trunk) or a multiple arterial phase (MAP) imaging technique (acquired with an 18s breath-hold 20s after the start of contrast media injection and further reconstructed with a temporal resolution of 3s).

For acquisition of ECA-MRI, 0.1 mmol/kg of extracellular contrast agent, including gadopentetate dimeglumine (Magnevist®; Bayer Schering Pharma AG), gadoterate meglumine (Dotarem®; Guerbet), or gadobenate dimeglumine (MultiHance®; Bracco), was injected at a rate of 2.5 ml/s. For acquisition of EOB-MRI, 0.025 mmol/kg of EOB (Primovist®; Bayer Schering Pharma AG or XianAi®; Chia Tai TianQing Pharmaceutical Co., Ltd) was injected at a rate of 1.0-2.0 ml/s.

External testing centers

For the external testing centers, five types of MRI scanners (Siemens MAGNETOM Vida 3.0 T, Siemens MAGNETOM Skyra 3.0 T, Siemens MAGNETOM Prisma 3.0 T, Philips Achieva 1.5 T, and uMR780 3.0 T) were used for the acquisition of contrast-enhanced MR images. Both ECA and EOB were used. For the acquisition of ECA-MRI, 0.1 mmol/kg of gadopentetate dimeglumine (Magnevist®; Bayer Schering Pharma AG, Berlin, Germany or SCHERING, Guangzhou, China) or gadoterate meglumine (Dotarem®; Guerbet) was injected at a rate of 2.5 ml/s. For the acquisition of EOB-MRI,

0.025 mmol/kg of EOB (Primovist®; Bayer Schering Pharma AG, Berlin, Germany or XianAi®; Chia Tai TianQing Pharmaceutical Co., Ltd, Jiangsu, China) was injected at a rate of 1.0-2.0 ml/s.

The MRI sequences included (a) T2-weighted imaging; (b) diffusion-weighted imaging (b values: 0, 60, and 800s/mm^2 [Siemens MAGNETOM Vida 3.0 T]; 0, 50, 400, and 800s/mm^2 [Siemens MAGNETOM Skyra 3.0 T]; 0, 20, 50, 100, 150, 200, 600, 1000, 2000, and 3000s/mm^2 [Siemens MAGNETOM Prisma 3.0 T]; 0, 50, and 600s/mm^2 [Philips Achieva 1.5 T]; 0, 50, and 1000s/mm^2 [uMR780 3.0 T] with apparent diffusion coefficient maps reconstructed using the monoexponential model; (c) in- and out-of-phase T1-weighted imaging; and (d) dynamic T1-weighted imaging in the pre-contrast phase, late arterial phase, portal venous phase (60s after the start of contrast media injection), delayed phase (for ECA-MRI, 3min after start of contrast media injection) or transitional phase (for EOB-MRI, 5min after the start of contrast media injection), and hepatobiliary phase (for EOB-MRI, 20min after the start of contrast media injection).

Details of the MRI sequences and parameters are shown in **Table S1**.

Table S1 MRI sequences and parameters

Sequence	T1-weighted IP and OP imaging	Dynamic T1-weighted 3D GRE	T2-weighted 2D FSE	Diffusion-weighted imaging*
**Training and internal testing center: Siemens MAGNETOM Skyra 3.0 Tesla (18-channel body array coil)**				
Repetition time (ms)	81	3.95	2160	5600
Echo time (ms)	2.72/1.4	1.92	100	68
Echo spacing (ms)	/	/	8.3	0.7
Echo chain length	/	/	25	49
Number of echoes	2	1	1	1
Flip angle (°)	70	9	160	90
Section thickness (mm)	6	2.5	6	6
Spacing (mm)	1.8	-	1.8	1.8
Matrix size	352×286	352×256	320×288	100×76
Field of view (mm^2)	400×325	400×296	433×433	380×289
Acquisition time (s)	24	14	36	233
Fat suppression	No	Yes	Yes	Yes
**Training and internal testing center: Siemens Avanto 1.5 Tesla (30-channel body anterior coil)**				
Repetition time (ms)	72	5.41	2530	3600
Echo time (ms)	4.92/2.22	2.39	84	88
Echo spacing (ms)	/	/	7.6	0.69
Echo chain length	/	/	29	/
Number of echoes	2	1	1	1
Flip angle (°)	70	10	150	90
Section thickness (mm)	7	2.5	7	7
Spacing (mm)	7.8	-	7.8	7.8
Matrix size	256×187	320×152	256×187	192×115
Field of view (mm^2)	300×370	287×417	326×380	277×370
Acquisition time (s)	16	15	47	92
Fat suppression	No	Yes	Yes	Yes
**Training and internal testing center: Siemens TrioTim 3.0 Tesla (8-channel body anterior coil)**				
Repetition time (ms)	180	4.03	2700	5900
Echo time (ms)	3.67/2.2	1.39	95	76
Echo spacing (ms)	/	/	7.27	0.69
Echo chain length	/	/	29	/
Number of echoes	2	1	1	1
Flip angle (°)	65	9	140	90
Section thickness (mm)	6	2.4	6	6
Spacing (mm)	7.8	-	7.8	7.8
Matrix size	256×131	320×112	320×150	192×115
Field of view (mm^2)	295×450	250×500	272×464	294×393

Acquisition time (s)	18	17	RG	245
Fat suppression	No	Yes	Yes	Yes
**Training and internal testing center: GE Discovery 750w 3.0 Tesla (16-channel phased-array torsor coil)**				
Repetition time (ms)	150	4.1	6315	9230
Echo time (ms)	2.5/1.3	1.9	78	Minimum
Echo spacing (ms)	/	/	5.7	0.5
Echo chain length	/	/	28	/
Number of echoes	2	1	1	1
Flip angle (°)	70	15	111	90
Section thickness (mm)	6	2	6	6
Spacing (mm)	2	-	2	2
Matrix size	288×192	512×512	288×244	128 × 128
Field of view (mm^2)	420×420	380× 300	360×280	360× 380
Acquisition time (s)	31	15	RG	RG
Fat suppression	No	Yes	Yes	Yes
**Training and internal testing center: GE SIGNA™ Premier 3.0 Tesla (30-channel body anterior coil)**				
Repetition time (ms)	146.8	3.2	2200	5000
Echo time (ms)	2.3/1.1	1.4	85	Minimum
Echo spacing (ms)	/	/	6.7	0.5
Echo chain length	/	/	17	/
Number of echoes	2	1	1	1
Flip angle (°)	55	15	111	90
Section thickness (mm)	7	2.4	7	7
Spacing (mm)	2	-	2	2
Matrix size	320×192	320×240	320×224	120×240
Field of view (mm^2)	342×380	380×380	304×380	380×380
Acquisition time (s)	16	15	47	RG
Fat suppression	No	Yes	Yes	Yes
**Training and internal testing center: GE SIGNA™ Architect 3.0 Tesla (30-channel body anterior coil)**				
Repetition time (ms)	Auto	3.6	2100	Auto
Echo time (ms)	2.3/1.1	1.6	80	Minimum
Echo spacing (ms)	/	/	6.3	0.7
Echo chain length	/	/	28	/
Number of echoes	2	1	1	1
Flip angle (°)	55	15	111	90
Section thickness (mm)	7	1.5	7	7
Spacing (mm)	2	1.05	2	2
Matrix size	288×160	288×224	320×320	160×128
Field of view (mm^2)	380×380	380×380	380×380	380×380
Acquisition time (s)	Auto	15-17	45	RG
Fat suppression	No	Yes	Yes	Yes

**Training and internal testing center: Phillips Ingenia Elition X 3.0 Tesla (32-channel body anterior coil)**				
Repetition time (ms)	148	4.2	1000	1665
Echo time (ms)	2.4/1.2	1.49	90	56
Echo spacing (ms)	/	/	5.3	/
Echo chain length	/	/	33	53
Number of echoes	2	1	1	1
Flip angle (°)	50	10	90	90
Section thickness (mm)	6	1.6	6	6
Spacing (mm)	1.5	-	1.5	1.5
Matrix size	292×161	344×188	304×260	200×142
Field of view (mm^2)	380×282	380×281	380×380	380×301
Acquisition time (s)	12	14	24	152
Fat suppression	No	Yes	Yes	Yes
**Training and internal testing center: uMR588 1.5 Tesla (6-channel body anterior coil)**				
Repetition time (ms)	117.6	4.2	2600	3350
Echo time (ms)	4.7/2.27	1.88	99.2	77
Echo spacing (ms)	/	/	6.2	0.73
Echo chain length	/	/	31	45
Number of echoes	2	1	1	1
Flip angle (°)	60	10	90	90
Section thickness (mm)	6.5	2.5	6.5	6.5
Spacing (mm)	1.3	-	1.5	10
Matrix size	256×174	256×154	256×168	128×92
Field of view (mm^2)	320×400	255×400	427×320	320×400
Acquisition time (s)	29	13	39	RG
Fat suppression	No	Yes	Yes	Yes
**External testing center: Siemens MAGNETOM Vida 3.0 T (18-channel body array coil)**				
Repetition time (ms)	4.1	4.1	3000	6800
Echo time (ms)	1.31/2.54	1.31/2.54	92	59
Flip angle (°)	12	12	90	90
Section thickness (mm)	3.0	3.0	5	5
Spacing (mm)	0.6	0.6	1.25	1.25
Matrix size	320×203	320×203	320×74	140×112
Field of view (mm^2)	344×380	344×380	400×400	320×400
Acquisition time (s)	18	18	306	188
Fat suppression	No	Yes	Yes	Yes
**External testing center: Siemens MAGNETOM Skyra 3.0 T (18-channel body array coil)**				
Repetition time (ms)	81	3.95	2160	5600
Echo time (ms)	2.72/1.4	1.92	100	68
Flip angle (°)	70	9	160	90
Section thickness (mm)	6	2.5	6	6
Spacing (mm)	1.8	-	1.8	1.8
Matrix size	352×286	352×256	320×288	100×76

Field of view (mm^2)	400×325	400×296	433×433	380×289
Acquisition time (s)	24	14	36	233
Fat suppression	No	Yes	Yes	Yes
**External testing center: Siemens MAGNETOM Prisma 3.0 T (18-channel body array coil)**				
Repetition time (ms)	4.5	4.0	3000	3580
Echo time (ms)	2.5/1.1	1.7	75	56
Flip angle (°)	15	15	180	180
Section thickness (mm)	2.3	2.3	5	5
Spacing (mm)	0	0	1	1
Matrix size	320×177	320×180	256×256	128×88
Field of view (mm^2)	380×420	380×420	420×420	420×420
Acquisition time (s)	14	14	225	236
Fat suppression	No	Yes	Yes	Yes
**External testing center: Philips Achieva 1.5 T (8-channel body anterior coil)**				
Repetition time (ms)	90.7	3.9	1354	1144
Echo time (ms)	4.6/2.3	1.86	70	59
Flip angle (°)	80	10	90	90
Section thickness (mm)	7	4	7	7
Spacing (mm)	1	-	1	1
Matrix size	280×229	272×221	252×225	192×189
Field of view (mm^2)	375×304	375×295	375×298	375×302
Acquisition time (s)	18.3	16.7	120	126
Fat suppression	No	Yes	Yes	Yes
**External testing center: uMR780 3.0 T (12-channel body anterior coil)**				
Repetition time (ms)	147.9	3.4	2140	4000
Echo time (ms)	2.9/1.5	1.6	103.3	69
Flip angle (°)	70	10	100	90
Section thickness (mm)	6.5	4.5	6.5	6.5
Spacing (mm)	1.3	1.5	1.3	1.3
Matrix size	272×174	320×204	320×202	128×101
Field of view (mm^2)	400×320	400×300	380×300	380×300
Acquisition time (s)	32	15	228	147
Fat suppression	No	Yes	Yes	Yes

2D, two-dimensional; 3D, three-dimensional; FSE, fast spin-echo; GRE, gradient recall echo; IP, in-phase; MRI, magnetic resonance imaging; OP, opposed-phase; RG, respiratory gating.

[*]Images were acquired under free breath.

Supplementary Material 2: Assessment of image artifact

Radiologist 1 (with seven years of experience in liver MRI) assessed the degree of image artifact on the testing dataset with a five-point scale [1]: 1 = extensive artifact and images nondiagnostic; 2 = severe artifact but images still interpretable; 3 = moderate artifact with some but no severe effect on diagnostic quality; 4 = minimal artifact with no effect on diagnostic quality; 5 = no artifact. Score 5 was regarded as no artifact, while score 3 and 4 were regarded as minor artifact.

[1] Davenport MS, Viglianti BL, Al-Hawary MM et al (2013) Comparison of acute transient dyspnea after intravenous administration of gadoxetate disodium and gadobenate dimeglumine: effect on arterial phase image quality. Radiology 266:452-461

Supplementary Material 3: Image co-registration

Image co-registration ensured that the selected original MR images were consistent across all sequences. During image preprocessing, the images of different sequences were first registered to the portal venous phase images using a three-dimensional (3D) rigid registration network [2]. The multi-sequence liver registration network was used to establish spatial correspondence across different sequences of the same patient. The inputs of the registration network were the fixed and moving images, in which portal venous phase images were the fixed images and other images were the moving images. To improve the robustness and accuracy of the registration network, both the original images and the liver mask served as the inputs. The region of interest and initial displacement position for the registration network were roughly determined based on the liver mask. Finally, a linear transformation matrix was obtained as the spatial transformation relationship of the two input sequences (fixed and moving images) by the registration module. The registration accuracy was evaluated by the Dice coefficient and the average surface distance (ASD) between transformed moving label and the target label (**Table S2**).

[2] Yaniv Z, Lowekamp BC, Johnson HJ, Beare R (2018) SimpleITK Image-Analysis Notebooks: a Collaborative Environment for Education and Reproducible Research. J Digit Imaging 31:290-303

Table S2 The Dice and ASD results of 3D image registration network

	Dice	ASD (mm)
T2WI	0.891	3.27
DWI	0.883	3.61
IP/OP	0.907	3.08
Pre	0.932	2.32
AP	0.937	1.86
PVP	/	/
DP	0.945	1.71

Note.—3D, three-dimensional; AP, arterial phase; ASD, average surface distance; DP, delayed phase; DWI, diffusion-weighted imaging; IP, in-phase; OP, opposed-phase; Pre, pre-contrast T1-weighted imaging; PVP, portal venous phase; T2WI, T2-weighted imaging

Supplementary Material 4: Liver segmentation

The input liver mask, formed from liver segmentation, helped the model to pay more attention to the liver area and reduce information redundancy of original images. Liver segmentation was performed based on a well-established CNN framework, which has been described in detail in our previous publication [3]. In brief, the outline of the liver was automatically extracted from each sequence using a 3D segmentation framework to form the liver mask. In this study, a novel one-to-multiple unsupervised domain adaptation (OMUDA) framework, composed by a One-to-Multiple Domain Generation (OMDG) framework and a segmentation network, was proposed to segment multiple abdominal organs including liver from a range of MRI sequences by leveraging annotations of some existing annotated CT images, thereby avoiding the high cost of annotating target MR sequences. The whole framework is illustrated in **Figure S1**, and it consisted of two stages: CT-to-Multi-sequence MRI translation (Image Translation) and training with the translated target modality images (Segmentation). The former aimed to train a generator which translated a CT image to its corresponding MR images with multiple sequences simultaneously. The latter employed the generated multi-sequence MR images and the annotations of their corresponding source CT images to train segmentation networks, where marginal loss was used to fully utilize the samples from different datasets and handling this partial label learning problem. 3D MR images were fed into the model and the corresponding liver segmentations were obtained.

For implementation details, model hyperparameters were as follows: in the OMDG framework, the number of output branches of mapping network, style encoder and discriminator was 10, which equaled to the number of used sequences. Adam optimizer was utilized and the initial learning rates for the mapping network and other networks were 1e−6 and 1e−4, respectively. The batch size was 8 and the maximum iteration was set to 80000. Other hyperparameters were set the same as those in StarGAN v2 [4]. In the segmentation stage, 3D nnUNet [5] was used as the network structure in our study. Besides, the batch size was set to 8 and the nnUNet built-in postprocessing was discarded. The other settings were kept default.

The Dice Similarity Coefficient (DSC) and 95% Hausdorff distance (HD_{95}) were adopted to evaluate the segmentation performance of our OMUDA framework on the internal testing set (**Table S3**).

Comparison study was performed between our OMUDA and other state-of-the-art unsupervised synthesis frameworks: SIFA [6], CycleGAN [7], MUNIT [8] and StarGAN v2 [4] .The segmentation results of these methods in terms of Dice Similarity Coefficient (DSC) and 95% Hausdorff Distance (HD_{95}) are shown in **Figure S2**, which indicates that OMUDA outperformed SIFA, MUNIT and StarGAN v2 by a large margin for multiple organs (including liver) in multiple MRI sequences.

In addition, we assessed the robustness of our OMUDA segmentation networks on two external datasets: Multi-Modality Abdominal Multi-Organ Segmentation Challenge 2022 (AMOS22) and Combined Healthy Abdominal Organ Segmentation (CHAOS). OMUDA also achieved satisfactory

results that the average DSCs for multiple sequences and organs on the AMOS22 dataset and the CHAOS dataset were 82.66% (96.01% for liver) and 91.38%, respectively.

After image co-registration, the liver mask was similar across different sequences. Therefore, we only input liver mask from portal venous phase images into the model when developing CNN models using two- or three-sequence combinations. For one-sequence model, the corresponding liver mask of the target sequence was selected.

[3] Xu X, Chen Y, Wu J et al (2023) A novel one-to-multiple unsupervised domain adaptation framework for abdominal organ segmentation. Med Image Anal 88:102873

[4] Choi, Y., Uh, Y., Yoo, J., Ha, J.W., 2020. Stargan v2: Diverse image synthesis for multiple domains. In: 2020 IEEE/CVF Conference on Computer Vision and Pattern Recognition. CVPR.

[5] Isensee, F., Jaeger, P. F., Kohl, S. A., Petersen, J., & Maier-Hein, K. H. (2021). nnU-Net: a self-configuring method for deep learning-based biomedical image segmentation. Nature methods, 18(2), 203-211.

[6] Chen, C., Dou, Q., Chen, H., Qin, J., Heng, P.A., 2020. Unsupervised bidirectional cross-modality adaptation via deeply synergistic image and feature alignment for medical image segmentation. IEEE Trans. Med. Imaging 39 (7), 2494–2505.

[7] Zhu, J.Y., Park, T., Isola, P., Efros, A.A., 2017. Unpaired image-to-image translation using cycle-consistent adversarial networks. In: Proceedings of the IEEE International Conference on Computer Vision. pp. 2223–2232.

[8] Huang, X., Liu, M.Y., Belongie, S., Kautz, J., 2018. Multimodal unsupervised image-to-image translation. In: Proceedings of the European Conference on Computer Vision. ECCV, pp. 172–189.

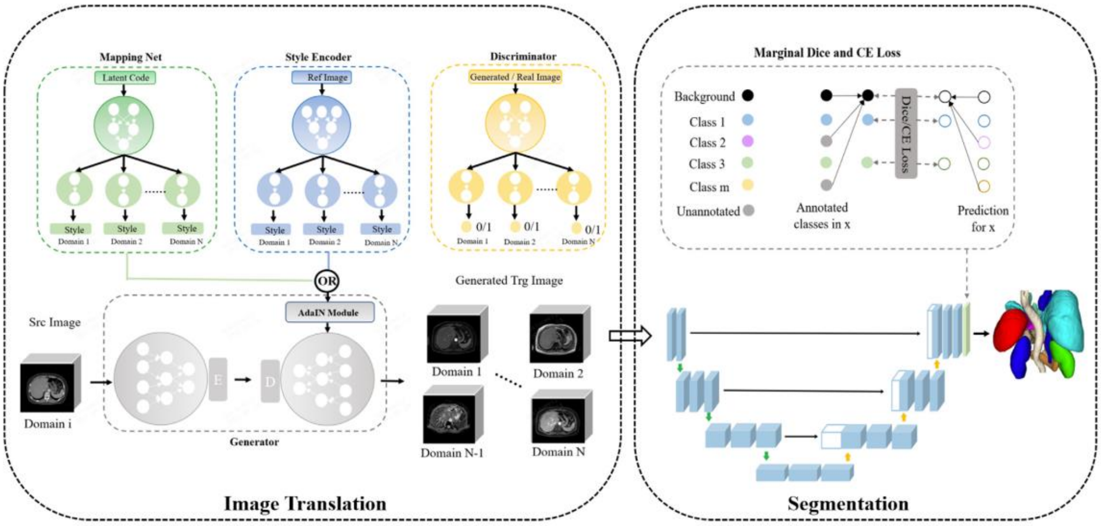

Figure S1 The one-to-multiple unsupervised domain adaptation (OMUDA) framework.

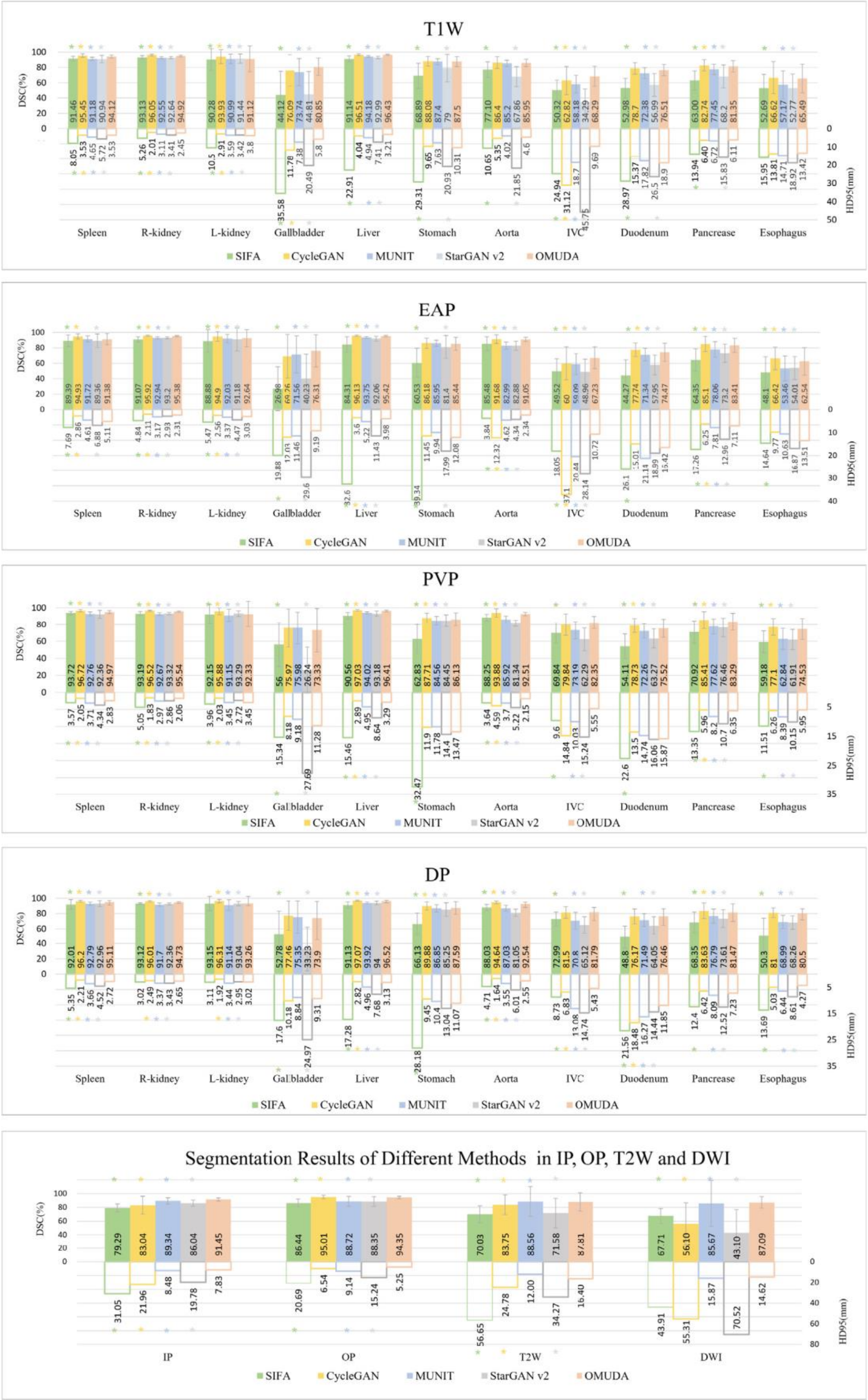

Figure S2 Segmentation results of different methods on the internal testing set.

Table S3 Quantitative liver segmentation results of OMUDA framework on the internal testing set

	DSC	HD_{95}(mm)
T2WI	0.878	16.40
DWI	0.871	14.62
IP	0.915	7.83
OP	0.944	5.25
Pre	0.964	3.21
AP	0.954	3.98
PVP	0.964	3.29
DP	0.965	3.13

Note.—AP, arterial phase; DP, delayed phase; DSC, Dice Similarity Coefficient; DWI, diffusion-weighted imaging; HD_{95}, 95% Hausdorff distance; IP, in-phase; OP, opposed-phase; Pre, pre-contrast T1-weighted imaging; PVP, portal venous phase; T2WI, T2-weighted imaging

Supplementary Material 5: CNN model training

Five-fold cross-validation strategy was applied to the training dataset during CNN training, in which the training dataset was further separated into derivation and validation sets for each fold. Five models were trained and the corresponding network weights with the best AUC value on the validation set were saved. The patients' predicted probabilities averaged from five selected models were used to calculate the evaluation metrics.

For CNN architecture, the filter size and stride for each convolution operation were the same as the standard ResNet-18 backbone (**Fig.1**), and the same padding strategy was used to ensure same spatial dimensions of the input tensor after convolution operation. One max pooling operation was performed after the first convolutional layer, and one average pooling was performed before the fully connected layer. The Kaiming initialization method was used to initialize the weights. Batch normalization and dropout strategy were used to tackle the challenges of long training time and overfitting, with batch size set to 32 and the dropout rate as 0.3.

During data loading of CNN training, balanced cirrhosis and non-cirrhosis samples were fed into the network to overcome the imbalance problem among two groups. The network was trained using the Adam optimizer with an initial learning rate (LR) of 1e-3, and the LR was updated by ReduceLRonPlateau schedule with a patience of 10 epochs and a decay factor of 0.1. Weight decay was set to 1e-5. Binary cross-entropy loss with label smoothing (delta=0.2) was applied as loss function. Batch size was set to 32 and dropout rate was 0.3. For above hyperparameter optimization, manual search technique was used during CNN training. The network was trained for a maximum of 500 epochs or until the early stopping condition had been met, storing the network weights with the best validation AUC value. In our study, early stopping with a patience of 20 epochs was performed when the AUC value on the validation set stopped improving, which was applied to further minimize overfitting.

During CNN training, learning curve was plotted to diagnose the overfitting over the experience of loss function (**Figure S3**). The loss curves of derivation and validation sets were almost parallel to each other, indicating that the CNN model generalized well on the validation set.

The averaged total training time of five-fold cross-validation experiments was 75 minutes, which was related to the model size, the number of workers during data loading, and other factors.

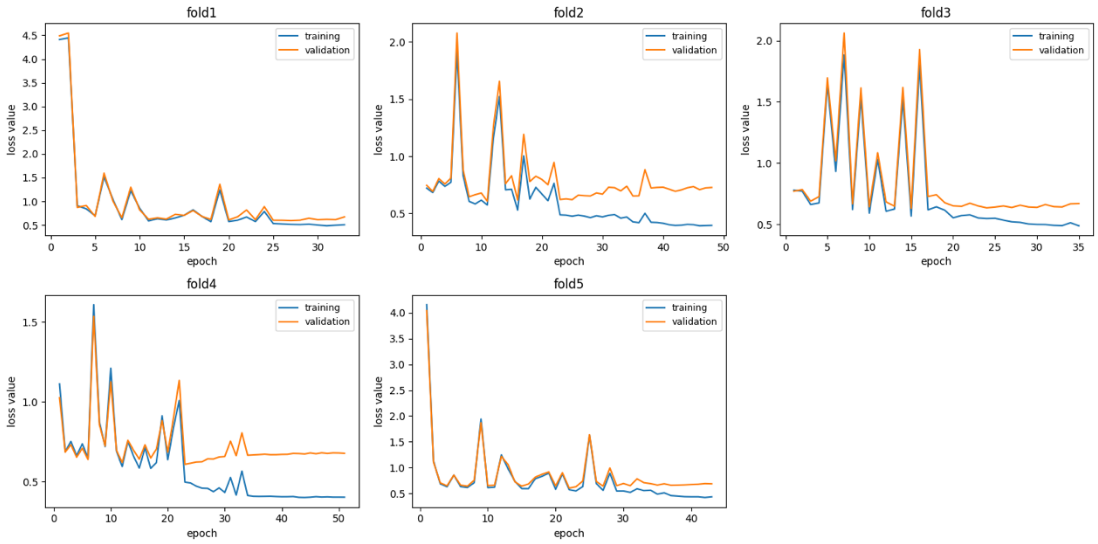

Figure S3 Loss curves of derivation and validation sets of five-fold cross-validation experiments.

Supplementary Material 6: Development of the combined model

Based on the training dataset, the combined model was established by integrating the CNN model with relevant clinical characteristics (i.e., age, sex, platelet count, total bilirubin, albumin, aspartate aminotransferase [AST], alanine aminotransferase [ALT], alkaline phosphatase [ALP], gamma-glutamyl transferase [GGT], international normalized ratio [INR], hepatitis B surface antigen, hepatitis B surface antibody, hepatitis B e-antigen, hepatitis B e-antibody, hepatitis B core antibody, hepatitis B virus status [present vs. absent], and hepatitis C virus status [present vs. absent]).

Table S4 Performance of CNN models with different combinations of MRI sequences on the training set

	Pre	T2WI	PVP	Pre + T2WI	Pre + PVP	T2WI + PVP	Pre + T2WI + PVP
Cut-off §	>0.43164	>0.53314	>0.42059	>0.54343	>0.45991	>0.31279	>0.38754
AUC	0.90 (0.88-0.92)	0.89 (0.86-0.91)	0.93 (0.91-0.94)	0.94 (0.92-0.96)	0.94 (0.92-0.96)	0.93 (0.91-0.95)	0.95 (0.93-0.96)
p value*	<0.001	<0.001	0.008	/	0.93	0.09	0.21
Sensitivity	82% (79%-85%)	82% (79%-85%)	82% (78%-85%)	87% (84%-90%)	84% (80%-87%)	87% (84%-90%)	83% (80%-86%)
p value*	0.002	0.009	<0.001	/	0.01	1.00	0.005
Specificity	84% (79%-88%)	81% (76%-85%)	90% (86%-93%)	89% (85%-92%)	92% (88%-95%)	85% (81%-89%)	93% (90%-96%)
p value*	0.009	0.001	0.85	/	0.11	0.06	0.02
PPV	90% (87%-92%)	88% (86%-90%)	93% (91%-95%)	93% (91%-95%)	95% (92%-96%)	91% (89%-93%)	95% (93%-97%)
p value*	0.002	0.001	0.99	/	0.12	0.04	0.03
NPV	73% (69%-76%)	72% (68%-76%)	74% (70%-77%)	80% (76%-83%)	76% (72%-79%)	79% (75%-83%)	76% (72%-79%)
p value*	<0.001	<0.001	<0.001	/	0.03	0.71	0.02
Accuracy	83% (80%-85%)	82% (79%-84%)	85% (82%-87%)	88% (85%-90%)	87% (84%-89%)	86% (84%-89%)	87% (84%-89%)
p value*	<0.001	<0.001	0.005	/	0.31	0.28	0.39
True positive	438	439	437	465	446	465	443
False positive	50	59	32	34	25	45	21
False negative	96	95	97	69	88	69	91
True negative	256	247	274	272	281	261	285

Note.—Data in parentheses are 95% confidence interval. AUC, area under the receiver operating characteristic curve; CNN, convolutional neural network; NPV, negative predictive value; PPV, positive predictive value; Pre, pre-contrast phase; PVP, portal venous phase; T2WI, T2-weighted imaging

§Cut-offs represent the model outputs of the CNN models.

**P* values were calculated in comparison to the model of Pre + T2WI. AUCs were compared using Delong test. PPVs and NPVs were compared using the weighted generalized score test proposed by Kosinski, while sensitivities, specificities, and accuracies were compared using McNemar's test.

Table S5 Multicollinearity test based on variance inflation factor

Variables	VIF
CNN model-based cirrhosis probability[§]	1.099
Age[§]	1.052
Sex	1.072
Platelet count[§]	1.149
Total bilirubin[§]	1.345
Albumin[§]	1.277
AST[§]	3.454
ALT[§]	3.439
ALP[§]	2.204
GGT[§]	2.144
INR[§]	1.433
hepatitis B surface antigen	1.966
hepatitis B surface antibody	1.429
hepatitis B e-antigen	1.294
hepatitis B e-antibody	1.442
hepatitis B core antibody	/
hepatitis B virus status (present vs. absent)	1.365
hepatitis C virus status (present vs. absent)	1.068

ALP, alkaline phosphatase; ALT, alanine aminotransferase; AST, aspartate aminotransferase; CNN, convolutional neural network; GGT, gamma-glutamyl transferase; INR, international normalized ratio; VIF, variance inflation factor

[§]Continuous variables were normalized with z-score.

Table S6 Importance score of variables in the combined models

Fold 1		Fold 2		Fold 3		Fold 4		Fold 5	
Variables	Importance score	Variables	Importance score	Variables	Importance score	Variables	Importance score	Variables	Importance score
Probability	0.43	Probability	0.32	Probability	0.35	Probability	0.60	Probability	0.46
Platelet count	0.13	Platelet count	0.15	Platelet count	0.15	Platelet count	0.10	Platelet count	0.13
INR	0.07	INR	0.09	INR	0.07	INR	0.04	INR	0.06
GGT	0.05	GGT	0.05	GGT	0.06	GGT	0.04	GGT	0.04
Albumin	0.05	Albumin	0.06	Albumin	0.06	Albumin	0.04	Albumin	0.06
ALP	0.05	ALP	0.05	ALP	0.05	Age	0.03	Age	0.04
Total bilirubin	0.04	Total bilirubin	0.05	Total bilirubin	0.05	Total bilirubin	0.03	AST	0.04
ALT	0.04	AST	0.05	AST	0.05	ALT	0.03	ALT	0.04

ALP, alkaline phosphatase; ALT, alanine aminotransferase; AST, aspartate aminotransferase; probability, convolutional neural network model-based cirrhosis probability; GGT, gamma-glutamyl transferase; INR, international normalized ratio

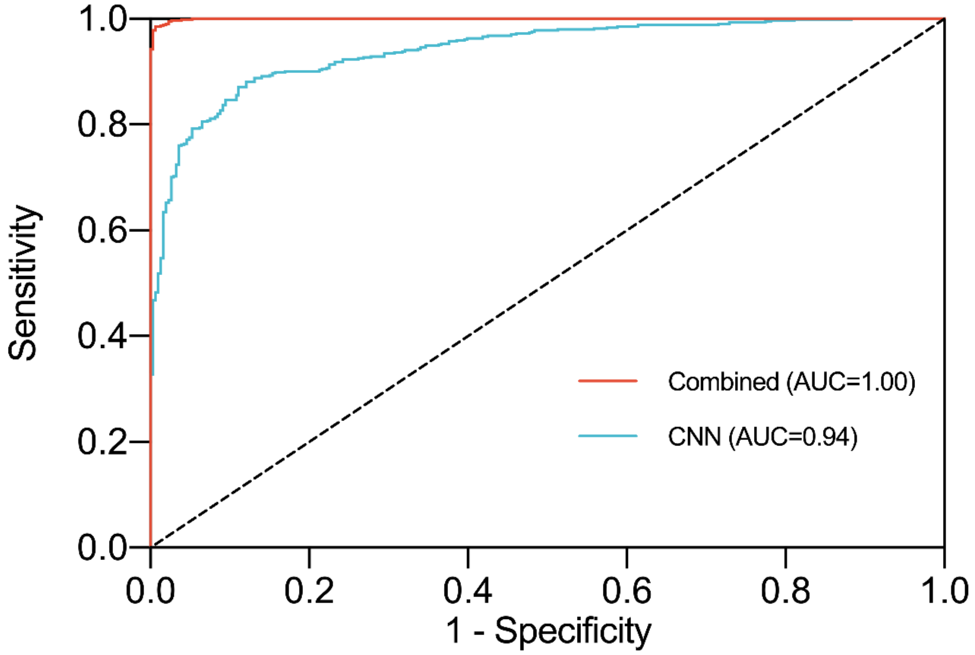

Figure S4 Receiver operating characteristic curves for diagnosis of cirrhosis on the training set. AUC, area under the receiver operating characteristic curve; CNN, convolutional neural network

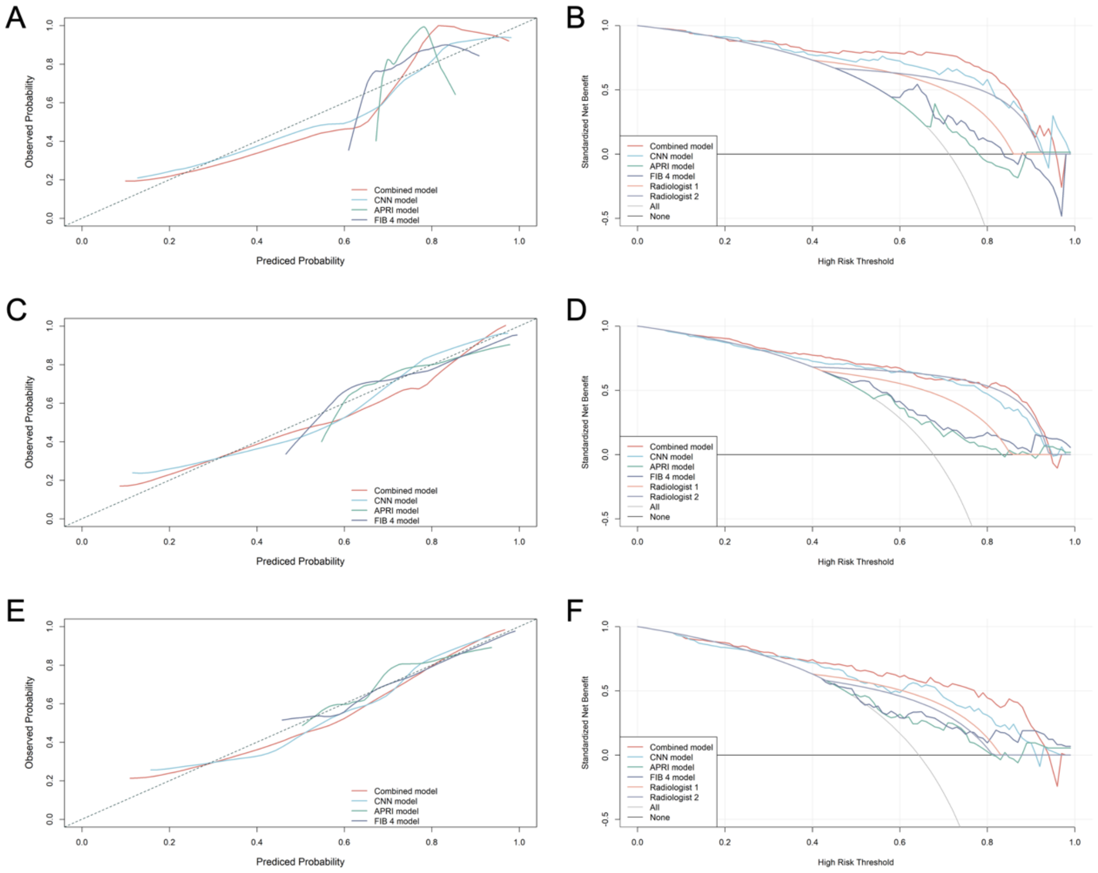

Figure S5 Calibration curves (A, C, and E) and decision curves (B, D, and F) for diagnosis of cirrhosis on the internal testing set 1 (A, B), internal testing set 2 (C, D) and external testing set (E, F). In decision curves, Y-axis is the net benefit, which describes the difference between the proportion of true positives and that of false positives weighted by the odds of the selected threshold for high-risk designation. X-axis represents the threshold probability. The threshold probability for a disease at which a patient decides to pursue treatment is the point where the expected benefit of treatment is equal to the expected benefit of forgoing treatment. At a given threshold probability, the model with the higher net benefit is preferred. APRI, aminotransferase-to-platelet ratio index; CNN, convolutional neural network; FIB-4, fibrosis-4 index

Table S7 Examples of possible reasons for false positive and false negative cases of the combined model on the testing sets

Cases	Activation maps of CNN model	Diagnostic results				FIB-4	APRI	PLT	Possible reasons
		CNN probability	CNN	Radiologist 1	Radiologist 2			(×10^9/L)	
False positive									
Case 1	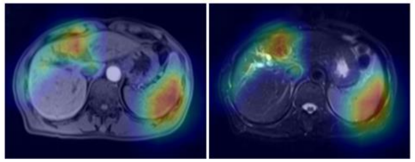	0.57	0	0	1	17.87	5.99	38	Platelet count decrease due to unknown reason
Case 2	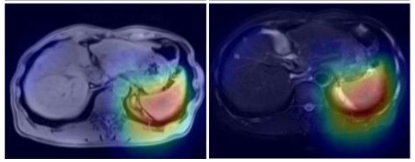	0.79	1	1	0	3.22	0.73	106	Unknown
Case 3	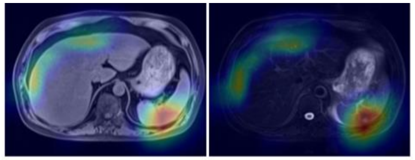	0.67	1	1	0	2.78	1.16	93	Slight liver surface nodularity on MRI
False negative									
Case 1	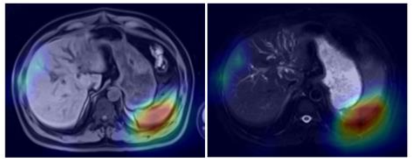	0.23	0	0	0	2.14	0.90	163	Lesion in left lobe leading to left lobe atrophy

Case 2	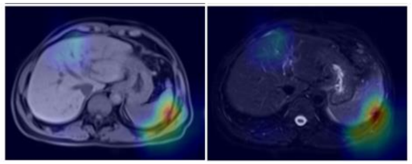	0.32	0	0	0	5.10	2.14	62	No significant imaging feature
Case 3	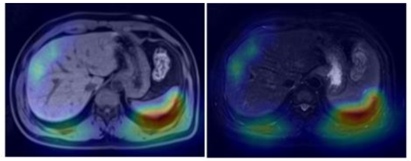	0.38	0	1	1	1.49	0.46	159	Unknown
Case 4	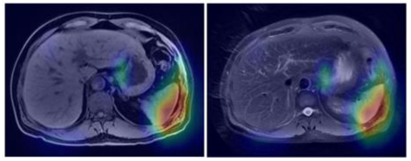	0.40	0	1	0	1.22	0.29	148	Unknown
Case 5	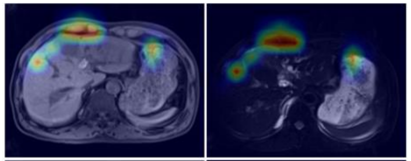	0.38	0	0	0	2.09	0.62	234	No significant imaging feature
Case 6	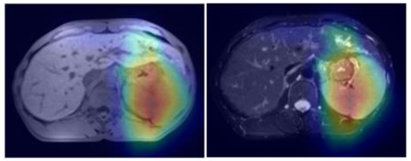	0.39	0	1	1	2.97	0.80	97	Unknown
Case 7	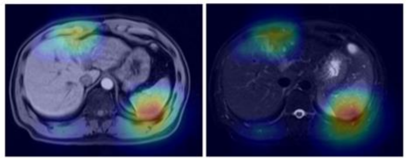	0.43	0	0	0	1.53	0.58	165	No significant imaging feature

Case 8	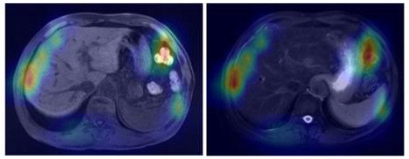	0.58	1	0	0	2.28	0.49	157	No significant imaging feature<br>Normal lab test results

APRI, aminotransferase-to-platelet ratio index; CNN, convolutional neural network; FIB-4, fibrosis-4 index; PLT, platelet count
